# Fast online spectral-spatial pulse design for subject-specific fat saturation in cervical spine and foot imaging at 1.5 T

**DOI:** 10.1007/s10334-024-01149-8

**Published:** 2024-02-17

**Authors:** Christian Karl Eisen, Patrick Liebig, Jürgen Herrler, Dieter Ritter, Simon Lévy, Michael Uder, Armin Michael Nagel, David Grodzki

**Affiliations:** 1 Institute of Radiology, University Hospital Erlangen, Friedrich-Alexander-Universität Erlangen-Nürnberg (FAU), Erlangen, Germany; 2 Magnetic Resonance, Siemens Healthcare GmbH, Erlangen, Germany; 3 MR Research Collaborations, Siemens Healthcare Pty Ltd, Melbourne, Australia; 4 Division of Medical Physics in Radiology, German Cancer Research Center (DKFZ), Heidelberg, Germany

**Keywords:** Dynamic RF pulses, Dynamic transmission, Pulse design, 1.5 T MRI, Fat saturation

## Abstract

**Objective:**

To compensate subject-specific field inhomogeneities and enhance fat pre-saturation with a fast online individual spectral-spatial (SPSP) single-channel pulse design.

**Methods:**

The RF shape is calculated online using subject-specific field maps and a predefined excitation k-space trajectory. Calculation acceleration options are explored to increase clinical viability. Four optimization configurations are compared to a standard Gaussian spectral selective pre-saturation pulse and to a Dixon acquisition using phantom and volunteer (*N* = 5) data at 1.5 T with a turbo spin echo (TSE) sequence. Measurements and simulations are conducted across various body parts and image orientations.

**Results:**

Phantom measurements demonstrate up to a 3.5-fold reduction in residual fat signal compared to Gaussian fat saturation. In vivo evaluations show improvements up to sixfold for dorsal subcutaneous fat in sagittal cervical spine acquisitions. The versatility of the tailored trajectory is confirmed through sagittal foot/ankle, coronal, and transversal cervical spine experiments. Additional measurements indicate that excitation field (B1) information can be disregarded at 1.5 T. Acceleration methods reduce computation time to a few seconds.

**Discussion:**

An individual pulse design that primarily compensates for main field (B0) inhomogeneities in fat pre-saturation is successfully implemented within an online "push-button" workflow. Both fat saturation homogeneity and the level of suppression are improved.

**Supplementary Information:**

The online version contains supplementary material available at 10.1007/s10334-024-01149-8.

## Introduction

Fat suppression is a crucial aspect of clinical MR imaging. Fat signal, which usually appears bright, can aggravate acquisitions by obscuring pathologies (e.g. edema, inflammation) or adding chemical shift artifacts [1, 2]. Furthermore, insufficient suppression of fat signal can impede the observation of water signal enhancement due to contrast agents in tissues where fat and water are mixed (e.g. breast, bone marrow) [3]. The importance of adequate fat saturation for various clinical applications is described in summary of the ISMRM workshop on fat–water separation by Hu et al. [4]. Some examples are the improved ability to identify lesions (e.g., hepatocellular adenomas versus fat-containing hepatocellular carcinomas), better tissue contrast, reduction of ghost artifacts and a better visualization of the myocardium. A variety of suppression techniques is available to serve different requirements. These are based on either the frequency or relaxation time difference of fat and water.

One approach is the application of a chemical shift selective pre-saturation pulse to saturate the signal of undesired tissue, such as fat [5]. However, the effectiveness of saturation can be influenced by the homogeneity of the excitation (B1) and main (B0) fields. This technique offers versatility and rapid application possibilities since it involves only adding a single pulse per excitation. In this work, a Gaussian pre-saturation pulse with a bandwidth of 200 Hz is chosen to represent this method. The choice of bandwidth determines vendor-independent sensitivity to B0 field offsets and thus potential undesired water excitation and insufficient fat saturation. The inhomogeneities might be reduced by 2nd-order shimming or additional hardware, such as local shim coils [6, 7], but the latter are not suited for large body regions.

The short tau inversion recovery (STIR) technique utilizes an inversion pulse followed by excitation after a specific inversion time determined by the fat's longitudinal relaxation time [8, 9]. Hence, theoretically no fat spins contribute to the final signal. This technique is less sensitive to B0 offsets, but results in mixed contrast, reduced SNR and increased minimal TR.

Fat–water separation methods produce water-only and fat-only images by exploiting the different phase evolutions of fat and water at specific echo times (TEs). The original Dixon technique [10] employed two TEs but did not account for main field inhomogeneities. Over the years, more advanced versions have addressed various issues, making fat–water separation appealing for diverse applications [11–14]. However, challenges such as increased scan time due to multiple echo acquisitions and the complexity of potential phase errors are still areas of ongoing research [15–17]. These challenges may currently lead to longer acquisition times or fat–water swaps. Additionally, fixed echo times impact the admissible ranges for resolution, contrast, or bandwidth.

Advanced pre-saturation pulse designs, such as binomial or spectral-spatial (SPSP) pulses, offer complex pulse profiles that can mitigate the impact of field imperfections and modulate excitation in a spatial manner to selectively excite fat tissue while leaving water unaffected (or vice versa) in the presence of individual field distributions [18, 19]. These approaches reduce reliance on B0 shim quality. Early versions were sensitive to main field inhomogeneities and often resulted in prolonged excitation pulses. Meyer et al. [18] introduced the first 2D version with selective excitation in one spatial and one frequency dimension, later expanded to 3D by Morrell and Macovski [20]. Zhao et al. [21] published a 4D SPSP approach compensating for B0 and B1 inhomogeneities, evaluating it through phantom measurements and one in vivo application to the knee using the spoke [22] and SPINS [23] trajectories at 3 T. Recently, Lévy et al. [24] presented a subject-specific fat saturation pulse design for chemical exchange saturation transfer using parallel transmission at 7 T for the brain. Employing multiple transmission coils and relatively simple B0 distributions (as found in the head), they enabled a spatially 3D online optimization based on a "universal" k_T_-points trajectory, tested on 12 volunteers. Yet, enabling online radio-frequency (RF) pulse calculation for high magnitude, complex field distributions as in the spine or other similar body regions, while ensuring clinical applicability (i.e., within a few seconds) as a "push-button" application remained unsolved.

We introduce an advanced fat pre-saturation pulse approach within a turbo spin echo (TSE) sequence. Our method, referred to as "SPSP fat saturation", utilizes a universally tailored trajectory for spatial frequency manipulation during excitation and enables fast online RF pulse optimization for single-channel transmission at 1.5 T. Importantly, this approach can be easily adapted to various sequences without increasing acquisition time. The RF fat saturation pulse is calculated based on acquired field maps and the predetermined excitation k-space trajectory. To determine subject-specific pulses, we utilize an interior point solver provided by Majewski [25], while also implementing additional acceleration options for RF calculation. Multi-slice SPSP fat saturation is performed in four configurations (with different acceleration factors) in phantom and volunteer measurements. The focus is on sagittal cervical spine imaging, but further measurements on sagittal foot/ankle and experiments in coronal and transversal orientation for cervical spine are also presented. A common Gaussian pre-saturation pulse is used for direct comparison of the pre-saturation techniques. In addition, Dixon measurements are performed to present the state-of-the-art in water-only images that can be interpreted as a target for the used pre-saturation methods.

## Methods

### Excitation k-space trajectory optimization

The selection and parameterization of gradients that are concurrently applied to the RF pulse, forming the excitation k-space trajectory [26], play a vital role in achieving an appropriate pulse design [21, 23, 27–31]. To address this, we conduct an offline trajectory optimization with six volunteer data sets prior to the scans and evaluate the resulting trajectory with six additional volunteer data sets (in total: 6 female, 2 diverse, median age 47 years, range 19–80 years). The data sets include information of the sagittal cervical spine. All in vivo MRI measurements are performed in accordance with institutional guidelines and all healthy volunteers provided informed consent prior to the examination. This optimization involves considering corresponding field maps, masks, scanner properties, and other relevant factors. The resulting trajectory parameterization is then utilized for subject-specific online calculations of the SPSP fat saturation RF pulse. The optimization process presented can be used as a method for improving existing parameter values of an already initialized trajectory, but also as a structured initial parameterization when creating a new trajectory. If a parameterized trajectory that is well suited to the demands (e.g. target application, region) already exists, this process can be skipped.

**Fig. 1:**
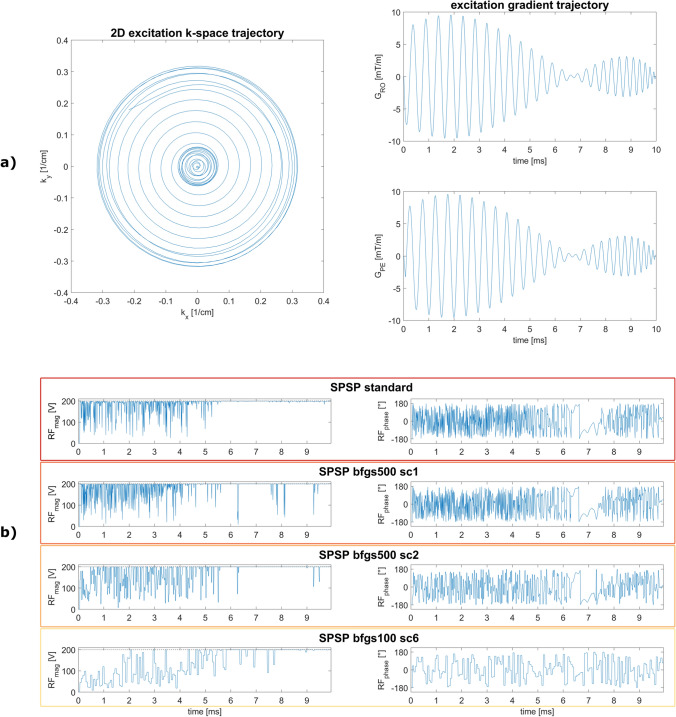
**a** Excitation k-space trajectory and gradients for the final parameter set. These parameters are optimized with six volunteer data sets (sagittal cervical spine) and ten initial points following MATLABs pattern search algorithm (part of the Global Optimization Toolbox in R2019b) and individual pulse calculations with subsequent Bloch simulations. The shown trajectory is used for all further experiments. **b** Complex RF pulse shapes for the proposed fat saturation method (SPSP) in standard and accelerated configurations for an exemplary volunteer measurement. The first column shows the RF magnitude and the second column represents the corresponding phase. As sampling compression factors (sc) increase, the RF pulse shape becomes more discretized

For the purposes of SPSP fat saturation focused on sagittal imaging of the cervical spine cord, a 2D spiral trajectory with six parameters, enabling in-plane frequency modulation through temporally varying radius and velocity in k-space, is employed. In contrast to a standard spiral trajectory [32], this implementation offers more flexibility to cover k-space and exploit scanner limits. In combination with a matching RF pulse, this trajectory aims to compensate for the existing field inhomogeneities and aims to provide uniform fat saturation without exciting water. A more detailed description of the optimization procedure and an illustration of the workflow can be found in the Supporting Information. The final trajectory is visualized in Fig. 1a, where $x$ and $y$ of the trajectory definition correspond to readout and phase encoding directions, respectively. The minimum spatial resolution of the trajectory is 3.13 cm for both directions. The inner spiral (which covers the last 4.2 ms) enables spatial variations with a resolution of 16.0 cm or more.

### RF pulse design

The RF pulse design process involves two phases: the configuration phase and the optimization phase. The configuration phase is performed offline and is the prerequisite of the subsequent online optimization phase.

Within the configuration phase, all settings and parameters required for the optimization procedure are determined. This includes defining the target flip angles (FAs) of 110° for fat (− 3.4 ppm) and 0° for water (0.0 ppm) [33], as well as the desired spatial pattern for both frequencies to achieve homogeneous (non-)excitation. The gradient trajectory is optimized beforehand as described in the previous section and the Supporting Information. The total pulse duration is determined by the number of trajectory samples, considering a sample duration of 10 μs. To ensure compliance with hardware long-term requirements, the RF voltage per sample is limited. In the standard SPSP configuration, an upper boundary of 100 iterations is set for the optimization algorithm. However, for two of the four presented SPSP fat saturation configurations, the number of iterations is increased to 500 to compensate for the potential accuracy loss introduced by the applied accelerated RF calculation approach discussed below. To reduce the size of the optimization problem, the resolutions of the field maps are scaled down by a factor of four.

The optimization phase represents the core element of the SPSP RF pulse design, which is based on an interior point solver [34, 35] implemented by Majewski [25]. In principle, this solver enables a simultaneous calculation of the RF pulse shape, sample duration, and gradient shape. For this work, the sampling duration and gradient shape are not determined during online optimization, but only the RF pulse shape. The optimization is based on explicit first and second order derivatives and aims to minimize the deviation between the resulting magnetization vector ${M}^{(n,f)}$ and a given target magnetization vector ${m}^{(n,f)}$ for all voxels $n\in (1,\dots ,{N}_{voxel})$ and frequency bands $f\in (1,\dots ,F)$ (here: $F= 2$) within a single slice using full Bloch simulations. The objective function for the optimization, as expressed in Eq. 1, minimizes the L2-norm of the difference between the magnetization vectors:

1 $${\text{min}}({\Vert {M}^{\left(n,f\right)}-{m}^{\left(n,f\right)}\Vert }_{2}^{2})$$

Please note that the sampling duration is set to 10 μs and the excitation k-space trajectory is optimized offline before the actual RF pulse calculation. Therefore, the minimization process in Eq. 1 pertains to the optimization of the complex RF shape only. This results in ${2}{\text{N}}_{\text{samples}}$ degrees of freedom (DOFs) [25], where ${\text{N}}_{\text{samples}}$ represents the number of RF pulse time samples to be optimized. For example, a pulse to be optimized with a total duration of 10 ms and sample duration of 10 μs consists of 1000 complex pulse samples and has a maximum of 2000 DOFs, independent from the number of voxels ${N}_{{\text{voxel}}}$. To extend the slice-specific optimization to a multi-slice application, neighboring slices are automatically compared according to the similarity of their B0 maps to identify slice blocks. Slices ${s}_{j}$ and ${s}_{i}$ (where $j,i\in \left\{1,\dots ,S\right\}$, $j\ne i$ with S the total number of slices) which are considered within one block need to fulfill both of the following conditions based on the voxel wise B0 difference $\Delta {B0}^{{s}_{j}{s}_{i}} = \sqrt{({B0}^{\left({s}_{j}\right)}-{B0}^{\left({s}_{i}\right)})^{2}}$ between voxels at the same in-plane position.

$$(1) \, {\text{mean}}\left(\left\{x\in \Delta {B0}^{{s}_{j}{s}_{i}}: x < 50\, {\text{Hz}}\right\}\right) < 20\, {\text{Hz}}$$

2 $$(2) \, |\left\{x\in \Delta {B0}^{{s}_{j}{s}_{i}}: x > 25\, {\text{Hz}}\right\}| < 0.3|\Delta {B0}^{{s}_{j}{s}_{i}}|$$

with $|x|$ denoting the cardinality of a given set. Threshold values are found empirically. These conditions ensure that the mean B0 difference for reasonable values ($< 50 {\text{Hz}}$) is below 20 Hz and that less than 30% of all values differ by more than 25 Hz. A common SPSP pulse is optimized for each slice block based on the center slice. The overall scheme of the TSE sequence remains unchanged, only the fat pre-saturation pulses are replaced by SPSP pulses. Neighboring slices within one block share the same SPSP pulse, but are acquired independently. For example, a measurement with 15 slices requires a maximum of 15 individual SPSP pulses. If five neighboring slices each have a similar B0 distribution, only three SPSP pulses need to be calculated. In the applied TSE sequence, these three pulses replace the standard fat saturation pulses of the corresponding slices, i.e. SPSP pulse #1 is applied to slices 1–5, SPSP pulse #2 is applied to slices 6–10, SPSP pulse #3 is applied to slices 11–15. Due to the selection of slice blocks for the pulse calculation, the overall preparation time is reduced. The algorithm is capable of minimizing the magnetization deviation by taking into account, among other aspects, B0 and B1 field inhomogeneities as well as system properties, such as the adjust transmitter voltage or the maximum applicable RF-voltage. The solver presented by Majewski, therefore, offers a well-fitting answer for the demands of a SPSP selective pulse design for online subject-specific fat saturation.

The actual implementation of the SPSP pulse design in an online workflow includes the following steps. After starting the TSE adapted with the proposed method, B1 and B0 mapping sequences are acquired. The results are processed (e.g. actual mapping, interpolation) and stored in binary MATLAB files together with the scanner properties. The actual pulse optimization uses this information and another file with the settings from the configuration phase to calculate the SPSP RF pulse in a MATLAB framework on the scanner computer as described in the optimization phase. The output of this optimization replaces the standard spectral fat pre-saturation pulse slice by slice.

To decrease the calculation time and enhance the clinical acceptance of the proposed fat saturation method, different acceleration techniques for RF calculation are developed. One technique involves treating ${N}_{{\text{sc}}}$ (sampling compression, sc) neighboring pulse samples as a single combined sample, resulting in a reduction of degrees of freedom (DOFs) by a factor of ${N}_{{\text{sc}}}$. This can be seen as down sampling the RF time axis. The resulting smaller optimization problem reduces the calculation time, with higher values of ${N}_{{\text{sc}}}$ providing stronger acceleration. Given that the number of RF pulse samples in our scenarios is 990, a configuration with a small ${N}_{{\text{sc}}}$ still offers sufficient DOFs for effective optimization. Another technique for accelerating the RF calculation is integrated into the interior point solver itself. In this approach, the second order derivatives are not explicitly determined but approximated based on the Broyden–Fletcher–Goldfarb–Shanno (BFGS) algorithm [36]. The application of BFGS is expected to require more iterations, but the individual iteration steps are performed faster compared to using the exact Hessians. These two acceleration techniques (RF time axis down sampling and BFGS) are independent of each other and can be applied simultaneously. Due to the trajectory setting, the optimization procedure uses ${\text{N}}_{\text{samples}}$ = 990, 445, 165 for SPSP configurations with different sampling compression factors.

To assess the impact of the proposed accelerated RF calculation approaches on calculation time and fat saturation quality, four different SPSP configurations are considered. The "SPSP standard" configuration does not employ any acceleration techniques and is limited to a maximum of 100 iterations. "SPSP bfgs500 sc1" and "SPSP bfgs500 sc2" introduce moderate acceleration by activating the BFGS approximation, combining one or two neighboring samples (${N}_{{\text{sc}}}$=1, ${N}_{{\text{sc}}}$=2), and allowing a maximum of 500 iteration steps. The fourth configuration, "SPSP bfgs100 sc6," employs the BFGS algorithm, a sampling compression factor of ${N}_{{\text{sc}}}$=6 and a maximum of 100 iterations. This configuration is expected to reveal the limitations of the proposed acceleration methods.

### Data acquisition and performance evaluation

All measurements are conducted on a 1.5 T whole-body system (MAGNETOM Sola, Siemens Healthcare, Erlangen, Germany) using a Head/Neck Coil with 20 receive channels. 2-point Dixon measurements (later referred to as Dixon) are performed to acquire fat/water images for comparison purposes. For the SPSP fat saturation experiments, B0 and B1 maps are obtained prior to the actual measurements using default settings provided by the vendor (additional time ≈ 30 s). The acquired maps are subsequently automatically masked to eliminate any background influences. A clinical T2-weighted TSE sequence is utilized with the standard fat pre-saturation pulse, referred to as "Gaussian fat sat," and the subject-specific SPSP pulse, which replaces the Gaussian pulse. More sophisticated pre-saturation approaches like SHARP pulses [37, 38] or B1-insensitive WET pulses [39, 40] have been presented in the past but are not available for this work. Therefore, the Gaussian pulse is taken as comparison to the proposed SPSP method. The SPSP pulse optimization serves as “push-button” application that does not involve any transfer to a networked computer, but is triggered automatically (including B0 and B1 acquisition) by starting the sequence on the scanner computer. The primary focus of this study is to assess the feasibility of the proposed method in sagittal cervical spine imaging, where significant changes in B0 along the slice direction are not expected. Consequently, non-selective SPSP pulses calculated based on single slices, as described earlier, are applied. Furthermore, experiments involving sagittal foot/ankle imaging and simulations for coronal and transversal cervical spine imaging are conducted to demonstrate the potential extension of the method to other body parts and image orientations. Additionally, the necessity of B1 information in sagittal cervical spine imaging is investigated. The protocol parameters for all utilized sequences are provided in Table 1. Since the proposed SPSP pulses directly replace the Gaussian pre-saturation pulse, TR remains unchanged.

**Table 1:** Acquisition protocol parameters for Dixon and TSE measurements

		TR (ms)	TE (ms)	TA (min:s)	FOV (mm^3^)	Resolution (mm^3^)	Slices
Phantom	Dixon	4000	77	2:18	275.0 × 275.0 × 61.4	1.1 × 1.1 × 2.0	28
	TSE		73	1:22			
Sagittal c-spine	Dixon	3500	93	3:21	220.0 × 220.0 × 49.2	0.7 × 0.7 × 3.0	15
	TSE		87	1:47			
Coronal c-spine	TSE						
Transversal c-spine	TSE						
Sagittal foot/ankle	Dixon	4000	77	4:10	150.0 × 300.0 × 61.4	0.6 × 0.6 × 2.0	18/28
	TSE		76	2:18			

Seven configurations are employed for the measurements, where SPSP pulses use the previously tailored universal trajectory based on in vivo data: (A) with no fat saturation, (B) with 2-point Dixon, (C) with Gaussian fat saturation, (D) with the proposed SPSP fat saturation standard setting ("SPSP standard"), (E/F) with two moderately accelerated settings ("SPSP bfgs500 sc1" and "SPSP bfgs500 sc2"), and (G) with a strongly accelerated setting ("SPSP bfgs100 sc6").

The phantom and volunteer data are evaluated based on the remaining signal and signal inhomogeneity within the selected regions of interest (ROIs). The metric for the "remaining signal" is determined by calculating the overall mean and standard deviation of the signal intensities from individual measurements. This value is ideally 0 for fat only regions and 1 for water only regions. The "inhomogeneity" is quantified by calculating the mean and standard deviation of the signal variations within the single measurements and ROIs. The lower this value is, the more homogeneous the considered ROI appears.

#### Phantom measurements

The phantom used in this study has a foot-like shape and consists of two liquid compartments representing water and fat tissue. The water compartment is filled with a nickel sulfate solution (1.25 g NiSO_4_ × 6H_2_O per 1000 g H_2_O), while the fat compartment contains MARCOL 82 oil mixed with MACROLEX blue dye (0.011 g MACROLEX blue per 1000 ml MARCOL 82 oil).

To simulate different acquisitions, all measurements are repeated three times, with adjustment invalidation and re-shimming performed between each run. For evaluation, fat and water ROIs are determined manually.

#### Volunteer measurements

We performed cervical spine (3 female, median age: 62 years, range 39–66 years) and foot/ankle (2 female, median age: 54 years, range 39–66 years) acquisitions in five healthy volunteers. All measurements are conducted in accordance with institutional guidelines and the volunteers provided informed consent prior examination. This sample size aligns with the median sample size typically employed in MRI technical development studies [41] and should be an adequate number to explore technical feasibility.

Three ROIs (subcutaneous fat dorsal, spine and subcutaneous fat ventral for spine; subcutaneous fat dorsal, heads of metatarsals and tibia distal for foot/ankle) are defined manually. For quantitative analysis the ratio to an image with no fat saturation reveals the relative residual signal. The imaging protocols and SPSP RF pulse optimization settings used for the phantom acquisitions (configurations (A)–(G)) are replicated to ensure comparability in the measurements.

## Results

### Phantom measurements

**Fig. 2:**
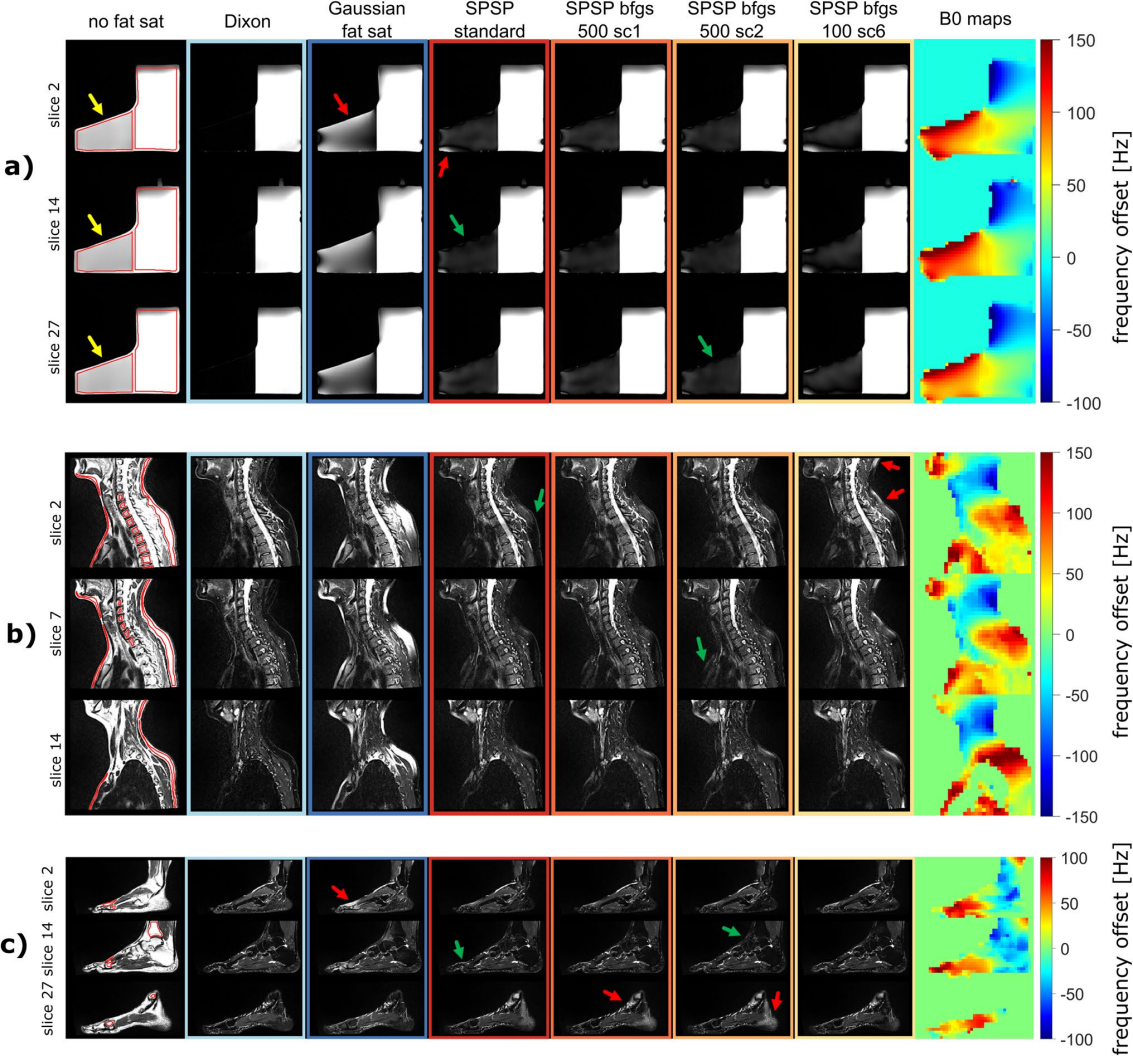
Multi-slice examples of phantom (1 slice block) (**a**), sagittal cervical spine (3 slice blocks) (**b**) and foot/ankle (4 slice blocks) (**c**) measurements with no fat saturation, Dixon, Gaussian fat sat and different SPSP configurations. The fat saturation quality achieved with Dixon is targeted with the pre-saturation approaches. **a** Fat ROI is highlighted with yellow arrows. Gaussian fat sat is not capable of saturating fat in presence of large B0 offsets, leading to insufficient fat saturation (red arrow). SPSP fat saturation improves fat signal suppression for all slices (green arrows). B0 inhomogeneities along slice direction do not show strong variations. **b** In vivo measurements comparing the same methods and settings used for phantom acquisitions. The SPSP fat pre-saturation shows a lower residual fat signal, especially in subcutaneous fat (green arrows). The configuration "SPSP bfgs100 sc6" differs from the less accelerated settings. This is mainly visible in the dorsal subcutaneous fat (red arrows). **c** In vivo foot/ankle measurements demonstrating applicability of the trajectory to other body parts. The fat saturation towards the toes (red arrow in Gaussian fat saturation) can be improved with the proposed method (green arrows). Inaccuracies in the B0 maps lead to incorrect fat saturation in higher slices (represented by slice 27, red arrows)

Three exemplary slices from one phantom data set comparing no fat sat, Dixon, Gaussian fat sat and the four SPSP configurations are shown in Fig. 2a. The same SPSP pulse is applied to all 15 slices (1 slice block determined). The fat region is highlighted with a yellow arrow. The corresponding B0 maps illustrate slight variations in the main field distribution along the slice direction. In-plane B0 offsets for all three data sets range between − 157 ± 59 Hz (minimum B0 offset) and + 199 ± 12 Hz (maximum B0 offset) for all slices and repetitions. In particular, strong frequency deviations occur in the fat region. In this region, the acquisitions with Gaussian fat saturation have a high proportion of residual signal, while all SPSP configurations appear darker. Dixon water images provide a qualitative representation of homogeneous and accurate fat saturation.

**Fig. 3:**
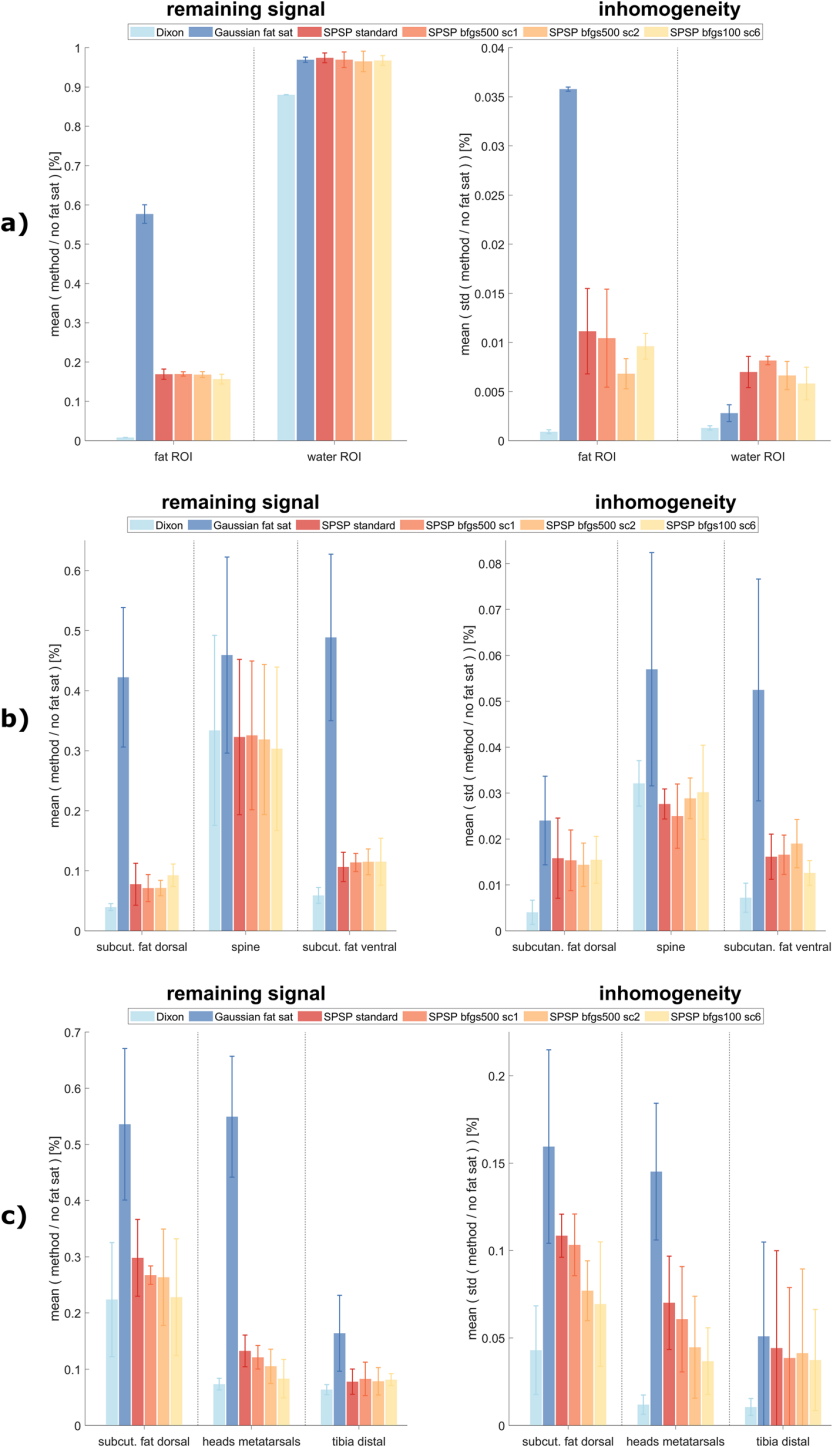
“Remaining signal” and “inhomogeneity” results for **a** phantom, **b** volunteer sagittal cervical spine and **c** volunteer sagittal foot/ankle measurements showing a clear improvement of fat saturation using the proposed SPSP approach compared to Gaussian fat sat in both metrics. Dixon indicates a state, where fat is saturated and water is not excited. For **a** Dixon has almost no fat signal left for the entire fat ROI. Water signal is only slightly excited for all pre-saturation approaches. Dixon shows some water excitation caused by the implementation of the vendor. Quantitatively no great differences between the SPSP approaches are visible. For **b** SPSP performs better than Gaussian fat sat in all considered ROIs, especially in subcutaneous fat. The analysis of the sagittal foot/ankle measurements (**c**) shows a clear improvement with the SPSP approach compared to the conventional Gaussian pulse in dorsal subcutaneous fat and at the metatarsal bones, which confirms the visual impression. Dixon outperforms the pre-saturation approaches in terms of fat saturation quality and homogeneity

In terms of Eq. 1, ${N}_{{\text{voxel}}}$ = 693 ± 11 voxels per slice are considered for phantom measurements. Quantitative analysis involves calculating ratios relative to the measurement with no fat saturation, utilizing the "remaining signal" and "inhomogeneity" metrics. All SPSP configurations show reduced overall signal below 18% in the fat ROI, whereas Gaussian fat sat still has nearly 60% remaining fat signal. Almost no fat signal is left with Dixon (< 1%). It should be noted that the Dixon water signal is approximately 10% lower than expected, likely due to the specific Dixon sequence provided by the vendor. All other techniques and configurations show similar results in the water ROI. Inhomogeneity for fat is reduced with the proposed SPSP approach. However, it is slightly increased for water. The results are presented in Fig. 3a.

The corresponding calculation times are provided in Table 2, highlighting a maximum reduction in calculation time by a factor of 8.5 (for the "SPSP bfgs100 sc6" configuration) compared to the SPSP standard settings. According to the quantitative analysis, the highly accelerated configurations (F, G) seem to provide a fast solution for strong fat saturation.

**Table 2:** Mean calculation time per pulse, excluding acquisition time for B0 and B1 maps (≈ 30 s), for the different SPSP fat saturation configurations for phantom and volunteer measurements. Depending on the variations in the B0 distribution in slice direction, a different number of pulses is determined per measurement (which proportionally increases the preparation time). Dixon and Gaussian fat saturation require no further preparation time

mean calculation time per pulse (s)	Phantom	Volunteer sagittal cervical spine	Volunteer foot/ankle
# slice blocks	1	1, 3	2, 3,4, 5, 6
Dixon	–	–	–
Gaussian fat sat	–	–	–
SPSP standard	34.9 ± 8.0	33.3 ± 3.9	25.7 ± 5.1
SPSP bfgs500 sc1	14.9 ± 0.2	13.7 ± 1.5	10.4 ± 0.8
SPSP bfgs500 sc2	10.9 ± 0.3	10.3 ± 1.1	8.4 ± 0.6
SPSP bfgs100 sc6	4.1 ± 0.2	3.5 ± 0.2	3.8 ± 0.3

### Volunteer measurements: sagittal cervical spine

Similar to the procedure of the phantom measurements, five volunteers are examined to compare the proposed fat saturation method with Gaussian fat pre-saturation and Dixon. An exemplary volunteer data set is presented in Fig. 2b. For this volunteer 3 slice blocks are determined. SPSP pulses are calculated based on slice 6 (applied to slices 1–10), slice 13 (applied to slices 11–14) and slice 15 (applied to slice 15). The main field inhomogeneities range between − 187 ± 32 Hz (minimum B0 offset) and + 296 ± 51 Hz (maximum B0 offset) for all slices and volunteers. The images illustrate insufficient fat saturation achieved with the Gaussian pulse, particularly in the subcutaneous fat and muscle areas, where large regions still exhibit high signal intensities. In contrast, all SPSP configurations result in stronger suppression of the fat signal across all slices (examples illustrated with green arrows). Slight differences can be observed within the SPSP configurations, such as in dorsal subcutaneous fat for configuration (*G*), highlighted with red arrows. After applying the SPSP fat saturation method, there is still residual signal from fat, albeit with low intensity, in comparison to Dixon. The B0 distributions along the slice direction exhibit similar patterns, primarily influenced by the anatomy of the subjects.

The optimization considers an average of ${N}_{{\text{voxel}}}$ = 650 ± 128 voxels (see Eq. 1) across all slices and volunteers. Pulse shapes for all investigated SPSP configurations are presented for one volunteer data set in Fig. 1b. Additionally, Table 3 displays the corresponding mean RF energy applied to sagittal cervical spine imaging for all five volunteers. The energy applied with SPSP is up to 65 times higher than that with Gaussian fat saturation.

**Table 3:** RF energy of Gaussian fat sat and the considered SPSP configurations for five volunteer data sets in sagittal cervical spine. Since no pre-saturation pulse is required for Dixon, no additional RF energy needs to be applied

	Mean RF energy (G^2^ μs)	Std RF energy (G^2^ μs)
Dixon	–	–
Gaussian fat sat	0.8	0.0
SPSP standard	50.8	3.2
SPSP bfgs500 sc1	50.9	3.2
SPSP bfgs500 sc2	45.3	2.9
SPSP bfgs100 sc6	34.8	3.0

For evaluation purposes, three manually selected ROIs (subcutaneous fat dorsal, spine, and subcutaneous fat ventral) are analyzed by setting the images in ratio to acquisitions without fat saturation. The results of the quantitative analysis for all volunteers and slices are presented in Fig. 3b. These observations are consistent with the exemplary data set. In subcutaneous fat regions, the proposed SPSP fat saturation approach demonstrates saturation that is up to six times stronger and more homogeneous compared to Gaussian fat saturation. In spine, the SPSP method yields a similar level of homogeneity with lower signal intensity. Since the lipid content is lower compared to subcutaneous fat, relatively higher relative signal intensities are expected. When considering only the SPSP fat saturation results, minimal differences are observed, with slightly higher relative residual signal intensity for the more strongly accelerated configuration in the spine and dorsal subcutaneous fat regions. In comparison to Dixon, the residual fat signal with SPSP is nearly double in subcutaneous fat and similar in the spine.

The additional preparation time required to calculate the SPSP fat saturation RF pulses for the volunteer measurements (Table 2, third column) is similar to those for phantom acquisitions, with a reduction due to the applied accelerated RF calculation techniques by a factor of up to 9.6.

**Fig. 4:**
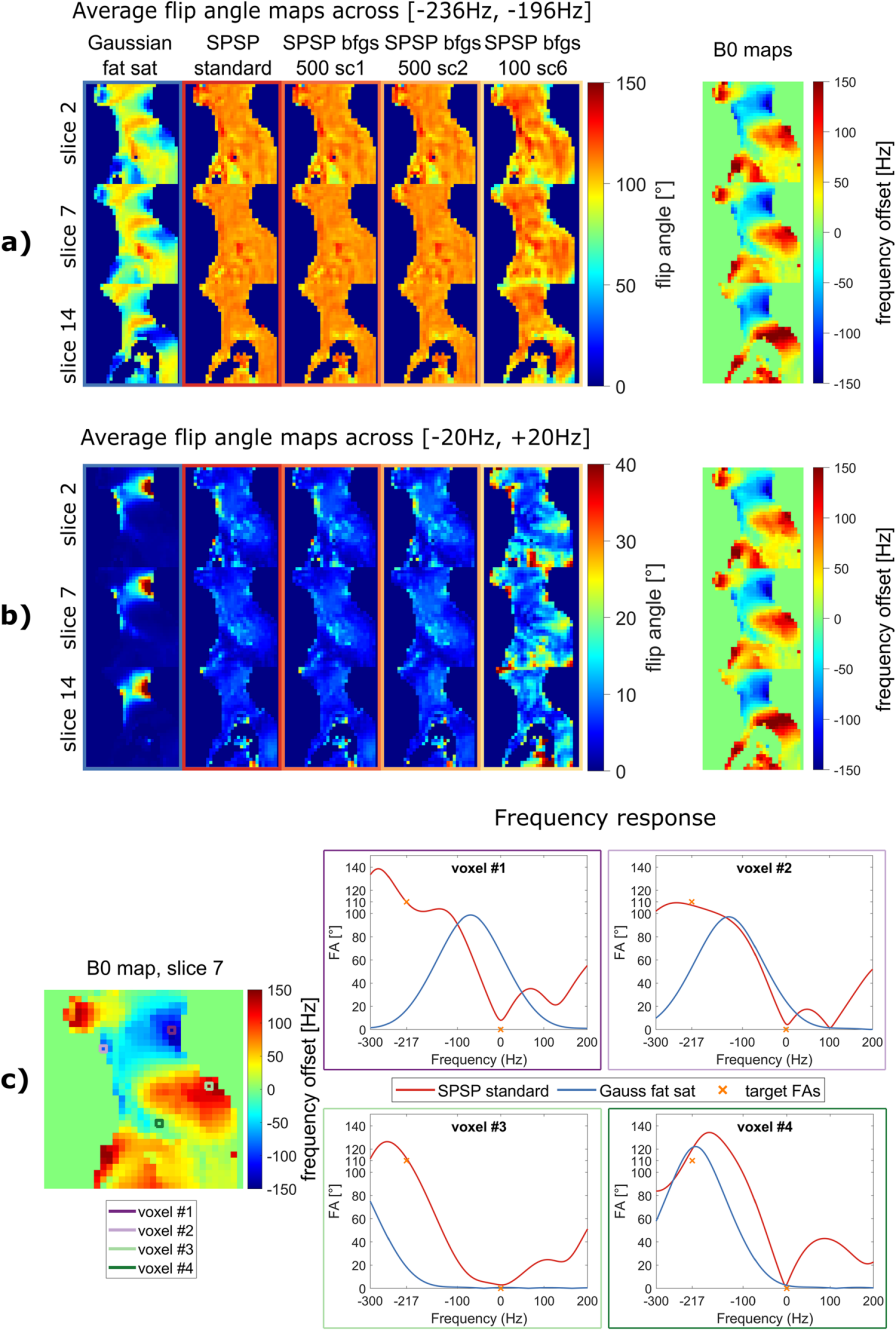
Simulated frequency response of Gaussian fat sat and different SPSP configurations at **a** fat and **b** water frequencies with a frequency range of ± 20 Hz for an exemplary volunteer data set. Strong deviations from the target FAs (110°/0°) are noticeable for Gaussian fat sat as a result of the corresponding B0 inhomogeneities. Therefore, unsatisfactory fat saturation and undesired water excitation are expected. Saturation quality of SPSP fat saturation decreases with stronger accelerated configurations for all slices, but still remains better than Gaussian fat sat in all settings. **c** Illustration of the specific frequency responses of four exemplary voxels showing that the Gaussian pulse is shifted along the frequency axis due to B0 inhomogeneities, while the SPSP pulses have location-dependent response curves responding to the field shifts. Yellow points mark the targets of the pulse optimization for fat and water, respectively

To investigate the stability of the pulses with respect to the accuracy of the B0 information and the assumed 1-peak fat model at − 3.4 ppm, Bloch simulations are performed and evaluated over different frequencies. The mean frequency responses of the target frequencies with a range of ± 20 Hz are shown in Fig. 4a) fat, b) water. In addition, the frequency responses of four exemplary voxels are shown in Fig. 4c. The frequency response of the SPSP pulses is location-dependent, which is why no overall response curves can be shown. The simulated flip angles for SPSP configurations (D)–(F) show consistent and homogeneous saturation around 110° for fat, with only slight excitation observed for water. In case of the strongly accelerated configuration (G), the expected pulse performance is slightly reduced but still outperforms Gaussian fat saturation.

### Volunteer measurements: sagittal foot/ankle

The tailored trajectory used in this study is primarily focused on sagittal cervical spine imaging. However, to assess the general applicability of the trajectory, sagittal foot/ankle SPSP fat saturation measurements are also conducted. Figure 2c shows an exemplary volunteer data set, demonstrating improvement in fat saturation. A comparison of the sequence configurations (A)–(D) for the center slice for all five volunteer data sets is presented in the Supporting Information. In this case 4 slice blocks are determined and SPSP pulses are calculated based on slice 5, 16, 24 and 27 (applied to 1–9, 10–21, 22–25 and 26–28, respectively). In particular, towards the toes, the quality of fat saturation enhances in all slices (green arrow in SPSP standard, slice 14) compared to Gaussian fat saturation (red arrow in Gaussian fat saturation, slice 2). However, in slice 27, there is more residual fat signal observed in the heel and towards the ankle with SPSP (exemplary red arrows in slice 27). It should be noted that these regions are not covered by the corresponding B0 map, while the distributions in the other presented slices are similar to the anatomical images. The example serves to demonstrate the general feasibility of the selected trajectory for other body parts. The sagittal foot/ankle measurements cover an average of ${N}_{{\text{voxel}}}$ = 195 ± 85 voxels (Eq. 1) for all slices of five volunteers.

Quantitative analysis similar to the analysis of the cervical spine images are conducted with three selected ROIs (subcutaneous fat dorsal, heads metatarsals, tibia distal) and presented in Fig. 3c. The remaining fat signal is up to sixfold lower with SPSP pulses compared to the Gaussian pulse. In addition, homogeneity of the remaining signal is improved with application of all SPSP configurations. Within the different configurations, “SPSP bfgs100 sc6” leads to the lowest remaining fat signal and the lowest inhomogeneity within the selected ROIs.

The mean preparation time for the five volunteers is provided in Table 2, last column. The most strongly accelerated SPSP configuration reduces the pulse calculation time by a factor of 6.8.

### Volunteer simulations: sagittal foot/ankle, coronal and transversal cervical spine

**Fig. 5:**
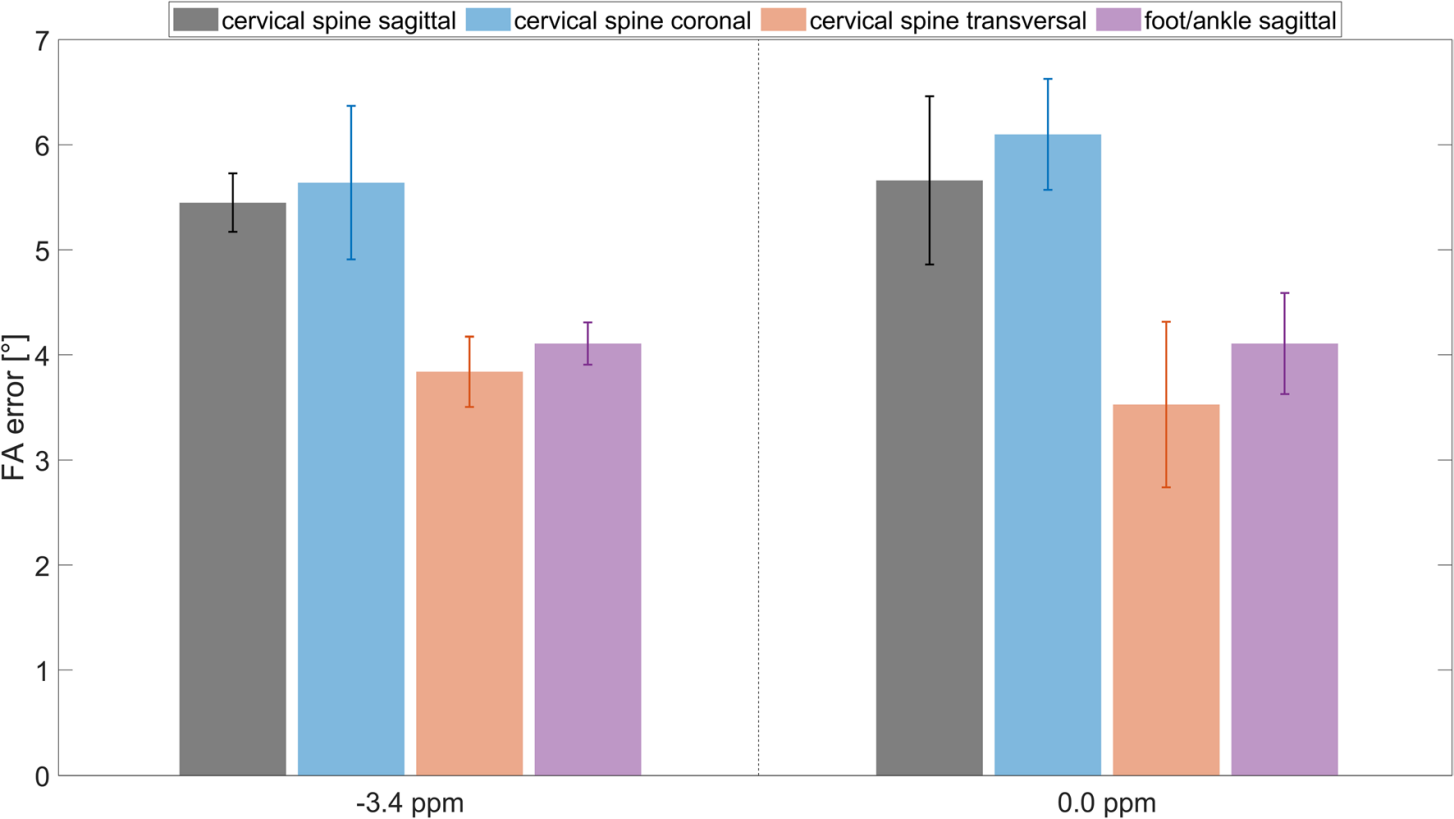
Flip angle error (average and standard deviation) of simulated FAs compared to target FAs (110°/0°) at fat (− 3.4 ppm) and water frequencies (0.0 ppm) for cervical spine sagittal, coronal, transversal and foot/ankle sagittal simulations based on five volunteer data sets. A universal application of the offline determined trajectory parameters based on sagittal cervical spine data sets to the presented body regions and orientations seems feasible

To evaluate the suitability of the excitation k-space trajectory for other image orientations and body regions, simulations are conducted based on volunteer data, including B0 and B1 maps. The simulations assess the deviations from the target flip angle (FA) at the fat and water frequencies for the sagittal, coronal, and transversal cervical spine, as well as the sagittal foot/ankle. The average deviations for these orientations are (5.4 ± 0.3/5.6 ± 0.7/3.8 ± 0.3/4.1 ± 0.2)° and (5.7 ± 0.8/6.1 ± 0.5/3.5 ± 0.8/4.1 ± 0.5)°, respectively (illustrated in Fig. 5). The number of voxels per slice for these simulation experiments are ${N}_{{\text{voxel}}}$ = 650 ± 128/572 ± 135/498 ± 191/195 ± 85. Note that actual measurements for orientations other than sagittal cervical spine and sagittal foot/ankle are not performed in this study. However, an outlook with measurements for the coronal foot/ankle and the transversal cervical spine is included in the Supporting Information.

### Volunteer measurements: impact of B1

**Fig. 6:**
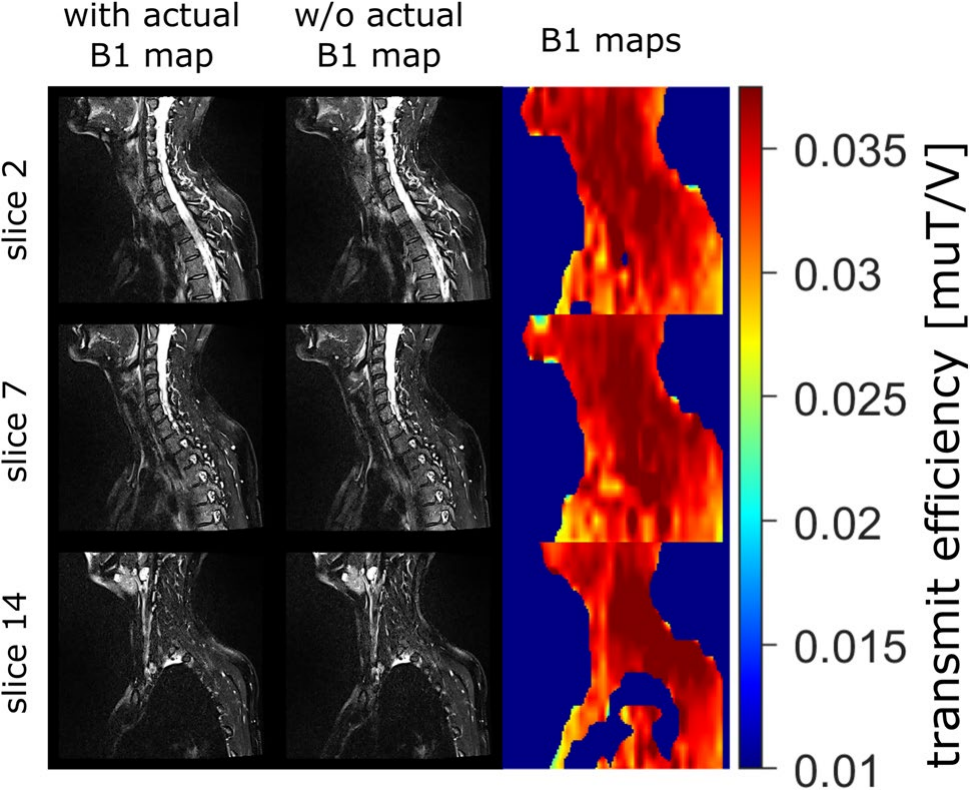
Exemplary multi-slice volunteer measurement of “SPSP standard” configuration considering the actual B1 map (1st column) and the nominal B1 value (2nd column) based on the adjust transmitter voltage. Results in dorsal subcutaneous fat and spine are similar, whereas ventral subcutaneous fat (especially in the chest area) shows slightly higher residual signal when the nominal B1 values is used. A similar B1 pattern is observed for all B1 maps, with lower values towards the abdomen, especially in the chest area

To investigate the impact of B1 inhomogeneities, sagittal cervical spine experiments with SPSP fat saturation including the actual B1 maps and pulse calculations with homogeneous B1 fields are conducted. The configuration is set according to “SPSP standard”. Exemplary results for one volunteer are presented in Fig. 6. For measurements with actual B1 maps, the mean relative signal percentages across five volunteers are (7.8 ± 3.5/32.3 ± 12.9/10.6 ± 2.4)% for dorsal subcutaneous fat, spine, and ventral subcutaneous fat, respectively. When utilizing SPSP pulse calculations based on the adjusted transmitter voltage, the mean relative signals are (8.2 ± 2.0/33.3 ± 12.7/15.3 ± 4.5)% for the same regions. The simulations indicate that ignoring the actual B1 distribution to design the SPSP pulse does not significantly deteriorate the actual pulse performance.

## Discussion

In this work, a fat pre-saturation approach is presented to improve the frequency selective fat saturation in presence of inhomogeneous fields, with focus on B0, by online subject-specific RF pulses. The method involves an offline optimization of the excitation k-space trajectory and an online calculation of individual RF pulses. The entire workflow is implemented on a single-channel 1.5 T system using a standard TSE sequence, which facilitates potential clinical translation.

The proposed technique is validated through phantom and volunteer acquisitions. In addition to sagittal cervical spine imaging, experiments are conducted for sagittal foot/ankle and coronal/transversal cervical spine orientations. The investigation also explores the necessity of B1 information for RF pulse optimization. Results from all measurements and simulations consistently demonstrate a significant improvement in terms of both remaining signal and homogeneity compared to the Gaussian fat pre-saturation method. However, it should be noted that the fat contrast achieved with the proposed SPSP method still does not match the contrast obtained using the 2-point Dixon method.

Phantom measurements provide insight into the weaknesses of simple spectral pre-saturation approaches (e.g. with a Gaussian pulse shape) for fat suppression in a reproducible setup. The results (Figs. 2a, 3a) demonstrate the sensitivity of these approaches to field inhomogeneities, leading to insufficient fat saturation and potential water excitation. Such issues can possibly lead to misinterpretation in clinical practice [42]. More advanced pre-saturation techniques, such as manually selecting the pulse shape with consideration of B0 and/or B1 inhomogeneities [37–40], or methods like Dixon, can overcome these limitations. However, these approaches come with their own drawbacks, such as prolonged acquisition time, limited contrast options, or potential phase swaps. Implementing Dixon in routine clinical practice requires individualized adaptations of sequences or protocols, e.g. by adding parallel imaging acceleration techniques [15–17, 43]. In contrast, the proposed dynamic SPSP method offers a solution with no need for complex sequence adaptations. By replacing the pre-saturation pulse with individually optimized RF pulses that counteract subject-specific field inhomogeneities, the obtained fat saturation is structurally improved compared to the conventional Gaussian fat pre-saturation in all measurements (Fig. 2b, c). Our quantitative analysis further demonstrates more accurate fat saturation using dynamically optimized SPSP, with residual fat signals up to six times lower compared to Gaussian fat saturation (Fig. 3b, c). In addition, water excitation in regions of clinical interest (e.g. spine) can be avoided. This effect can be observed in the phantom measurements presented (Fig. 2a), but also in the simulations for sagittal cervical spine images (Fig. 4b). For body regions or shims with stronger negative B0 offsets, stronger water excitation and insufficient fat saturation for a static fat pre-saturation pulses (i.e. the Gaussian pulse) can be expected. A severe case is presented in the Supporting Information for the transversal cervical spine. Due to the automatic selection of slice blocks for multi-slice acquisition, various SPSP pulses can be applied in different repetition times (TRs), with the risk of artefact formation. This risk increases the greater the B0 differences are in slice direction, as the SPSP pulse for slice ${s}_{j}$ could saturate the water signal in the neighboring slices ${s}_{i}$ (where $j, i\in \left\{1,\dots ,S\right\}$, S is the total number of slices). The calculation of the SPSP pulse of a slice block is based on the center slice. Therefore, the saturation quality in the other slices of the same block is expected to be lower than in the center slice. This effect is further investigated in the Supporting Information. Other approaches, such as calculation based on the average field distributions of all slices, could improve the performance. Adjusting the center frequency of the Gaussian pulse slice by slice is not expected to improve the result for this pulse, as the mean B0 offsets only differ by a few Hertz between slices (i.e. < 8 Hz for sagittal cervical spine and ankle/foot in vivo measurements). A broader bandwidth would cover more frequencies, resulting in better fat saturation, but at the price of stronger water excitation. However, the Gaussian pulse approach could benefit from adapting the center frequency and bandwidth to a volunteer-dependent average B0 offset and range. Dixon shows stable fat saturation results in phantom and volunteer measurements.

The optimization procedure for the excitation k-space trajectory in this work is a systematic and flexible approach that allows for the initialization or validation of parameter sets for different trajectories. Although the trajectory optimization is performed specifically for the sagittal cervical spine, the same trajectory settings are successfully applied to other targets and orientations, including phantom measurements, sagittal/coronal foot/ankle and transversal cervical spine acquisitions, and coronal/transversal cervical spine simulations. The results indicate that a single trajectory optimization for a region like the sagittal cervical spine can provide a sufficient solution for universal application. However, in more challenging cases, such as breast imaging, individual trajectory optimization may still be required. The maximum gradient strength used is 9.6 mT/m, which is below the capability of most systems. However, stronger gradient moments would only be necessary to reach areas further out in k-space and thus cover higher spatial frequencies. Since B0 inhomogeneities usually have low spatial frequencies, there is no need to use higher gradient moments. The velocity along the k-space trajectory (i.e. the revolutions of the spiral) is limited by the slew-rate, which exploits the given limits to the full (123.6 mT/m/ms, limit: 125.0 mT/m/ms). The parametrization of the proposed trajectory is more complex compared to most standard spiral trajectories [32]. This ensures a more flexible behavior, e.g. by non-equidistant revolutions and a varying sample density along the trajectory (see Supporting Information). Given the high slew rates, there may be other parameterized solutions that cover k-space in a similar way, but without the need for such fast gradient oscillations. The optimization procedure is applicable to all parametrized trajectories and is used to find optimal values for the parameters. However, there may be other trajectory designs or optimization schemes that provide a better foundation for the subsequent pulse design. A comparison of our proposed trajectory with a standard spiral trajectory and a spline-interpolated trajectory is presented in the Supporting Information. Previous work mostly focused on systems with multiple transmission channels, while our optimization is designed for single-channel systems. Some methods determine the trajectory parameter set analytically or by optimizing individual parameters while holding others fixed [23]. Another method is the simultaneous RF pulse and gradient optimization, which is common for k_T_-points or spokes [28, 29]. The approach of Herrler et al. [30] similar to the method presented in this work, but takes into account local specific energy dose restrictions and is applied to a 3D SPINS trajectory [23].

The objective of the presented SPSP method is to improve fat saturation with a subject-specific pulse design. A main aspect for this is a short preparation time. Currently, this includes the acquisition of field maps and the actual RF pulse calculation. The proposed acceleration methods for RF calculation can reduce the calculation time per pulse to less than 4 s (Table 2), while the pulse performance decreases only slightly. SPSP fat saturation determines slice blocks as a function of B0 variations in slice direction and empirical thresholds. Further reduction of preparation time could be achieved by optimizing these thresholds, resulting in fewer pulses to be calculated. Another aspect is the neglect of B1 information, as the quality of fat saturation does not seem to be significantly reduced. Since the actual B1 map is replaced by a homogeneous B1 map, no significant reduction in pulse calculation time is expected. However, the acquisition of the B1 map is omitted, which reduces the preparation time. Current approaches to fat pre-saturation are often combined with advanced shimming techniques that require additional time. The proposed method may eliminate this step. By combining all of the above, the presented SPSP approach could require almost no additional time compared to currently used fat pre-saturation techniques.

The RF pulse optimization in this work is based on an algorithm described by Majewski [25]. We have extended his work by incorporating additional acceleration methods for RF calculation and implementing the optimization process in an online "push-button" workflow directly at the scanner. Furthermore, we have investigated the potential of a universal trajectory optimization based on volunteer data, allowing for its application to various body parts and orientations. Previous SPSP approaches [21, 24, 44] focused primarily on higher field strengths (3/7 T), different applications, and sequences. For example, Zhao et al. presented a 4D scaled small-tip-angle approach using a spoke [22] or SPINS [23] trajectory, which was evaluated in a single in vivo measurement on the knee. Lévy et al. focused on a 7 T pTx system with a k_T_-points [45] and SPINS trajectory for fat saturation and water excitation for CEST imaging in the head, where B1 inhomogeneities were the main concern. In principle, these approaches could be transferable to our proposed workflow, considering the differences in sequence, system, body parts, and optimization parameters.

The pulse calculation in our method primarily relies on the measured B0 map and aims to achieve homogeneous flip angle distributions of 110° for fat (− 3.4 ppm) and 0° for water (0.0 ppm). Thus, it is essential to have reliable B0 information and a certain excitation bandwidth. In our study, the B0 map is obtained using a three-TE mapping approach offered by the vendor, which takes into account the chemical shift between fat and water. The spectrum in human adipose tissue is more complex than a simple 1-peak model [46] as assumed for the proposed pulse calculation. The dynamic SPSP pulses, along with the simultaneously applied gradients, create location-dependent voxel-wise frequency responses. Therefore, it is not possible to specify a global bandwidth. Local frequency responses of four exemplary voxels show the dynamic behaviour of SPSP pulses targeted at the specified frequency and flip angle combinations. Due to local frequency shifts, these global targets cannot be met by a simple Gaussian pulse. The analysis in Fig. 4 demonstrates the tolerance of SPSP to imperfect B0 information within the considered frequency range.

There are several further aspects to be investigated within the proposed method. One such aspect is the development of universal or FOCUS pulses [30, 47], which could further reduce the online computation time or potentially eliminate it completely. The focus of this work is on individually designed SPSP pulses based on a tailored trajectory. However, a brief outlook in form of a comparison with a simple universal pulse approach is presented in the Supporting Information. The RF energy of SPSP pulses is significantly higher compared to Gaussian pulses. Since the total RF energy in a TSE is usually dominated by refocusing pulses, we have not encountered any problems using the proposed approach at 1.5 T systems. However, in the current implementation without further restrictions besides a maximum peak voltage and pulse duration, this could be a limiting factor for higher field strengths. Nevertheless, we believe that the pulse design with specific RF energy considerations can be transferred to other field strengths (e.g. 3 T) without modifying the sequence in which it is applied (i.e. TSE). The adaptation and testing of the proposed method for other field strengths is not yet performed and is pending. The accuracy of SPSP fat saturation also relies on the simultaneous application of RF and gradient waveforms. Delays or deformations in the trajectory can significantly affect the quality of fat saturation. Additional gradient correction techniques could be employed to ensure proper timing of the RF and gradient samples [48–50]. Furthermore, extending the 2D pulse calculation to 3D has the potential to enhance the clinical applicability of the proposed technique. Suitable trajectories, e.g. based on SPINS, are already widely used for subject-specific pulse design [23, 30, 51, 52]. However, optimizing a larger number of voxels in a 3D setting while maintaining similar pulse lengths and degrees of freedom (DOFs) presents a challenge. The general transferability of the performance of the proposed method to other anatomies and orientations depends on various factors, such as the magnitude and spatial distribution of the corresponding B0 fields, the selected pulse length and the trajectory settings. Since the trajectory is the basis for the pulse calculation, a proper selection is crucial for the quality of fat saturation. We assume that more challenging areas require specially adapted trajectories for the individual purpose, e.g., an appropriately adjusted trajectory for SPSP fat saturation in breast imaging. In addition, longer SPSP pulses increase the DOFs and thus performance.

## Conclusion

We presented an online subject-specific fat pre-saturation method to compensate for field inhomogeneities for single-channel 1.5 T systems. The proposed approach is compared in four configurations with a standard Gaussian spectral selective fat pre-saturation pulse and a standard 2-point Dixon acquisition. The universal offline tailored trajectory is applicable to various body parts and image orientations. Phantom experiments demonstrate promising performances, which are confirmed in five volunteers on cervical spine and foot/ankle. With regards to both homogeneity and level of fat suppression, SPSP fat pre-saturation outperforms the Gaussian fat pre-saturation pulse (e.g., remaining fat signal of (42.2 ± 11.6)% with the Gaussian pulse compared to (7.2 ± 2.3)% with SPSP bfgs100 sc1 in dorsal subcutaneous fat in sagittal cervical spine measurements). Overall, this technique has the potential to enhance clinical imaging using fat pre-saturation in single-channel 1.5 T systems.

### Supplementary Information

Below is the link to the electronic supplementary material.

Supplementary file1 (DOCX 12434 KB)

## Data Availability

The data that support the findings of this study are available from the corresponding author upon reasonable request.
