# Supplementary material for: Fast online spectral-spatial pulse design for subject-specific fat saturation in cervical spine and foot imaging at 1.5 T
Source: MAGMA. 2024 Feb 17;37(2):257–72. doi: 10.1007/s10334-024-01149-8 (PMC10995033; doi:10.1007/s10334-024-01149-8)
Supplement: Supplementary file 1 — Supplementary file1 (DOCX 12434 KB) [file 10334_2024_1149_MOESM1_ESM.docx]

***Supporting Information***

**Fast online spectral-spatial pulse design for subject-specific fat saturation in cervical spine and foot imaging at 1.5 T**

Christian K. Eisen^1^, Patrick Liebig^2^, Jürgen Herrler^2^, Dieter Ritter^2^, Simon Lévy^3^, Michael Uder^1^, Armin M. Nagel^1,4^, David Grodzki^1,2^

**Affiliations**

^1^ Institute of Radiology, University Hospital Erlangen, Friedrich-Alexander-Universität Erlangen-Nürnberg (FAU), Erlangen, Germany

^2^ Magnetic Resonance, Siemens Healthcare GmbH, Erlangen, Germany

^3^ MR Research Collaborations, Siemens Healthcare Pty Ltd, Melbourne, Australia

^4^ Division of Medical Physics in Radiology, German Cancer Research Center (DKFZ), Heidelberg, Germany

**Corresponding author**

Christian K. Eisen

Universitätsklinikum Erlangen, Radiologisches Institut

Maximiliansplatz 3

91054 Erlangen, Germany

Office: +49(0) 9131 85-36276 | Email: christian.eisen@uk-erlangen.de

# Excitation k-space trajectory optimization

## Proposed trajectory configuration and optimization procedure description

The way of covering the excitation k-space is the foundation for the online subject-specific pulse calculation. Therefore, the parameters for the selected 2D spiral trajectory are optimized offline with 12 volunteer data sets (6 female, 2 diverse, median: 47 years, range: 19-80 years) focusing on sagittal view of the cervical spine, since this is the body region of main interest for this work. Six data sets are used for actual optimization according to the procedure presented below. The final parameter set for the trajectory is evaluated with six test data sets, which have not been included in the optimization procedure. Thus, a “universal” trajectory parametrization is found and used for all following experiments.

The trajectory’s parameter set (referred to as combined optimization values (COV), as implemented in by Herrler et al. [1]) consists of six parameters that describe the exact sample distribution in k-space and hence the accessible varying frequency space:

$COV :=[n_{1}, n_{2}, a, s, v_{1}, v_{2}]$

**Supporting Information Eq. S 1**

where $n_{1}{, n}_{2}$ define nodes determining the varying radius, *a* the covered fraction of k-space, *s* the number of samples (defining trajectory and pulse duration) and $v_{1}$, $v_{2}$ are factors determining the alternating velocity along the trajectory (influences of the single parameters on the trajectory are illustrated in Supporting Information Fig. S 1). These parameters describe a spiral trajectory that samples a certain fraction of the excitation k-space with flexible sample density towards the center and along the trajectory. Hereby, the radius of the trajectory samples is defined via 1D spline interpolation of [0, 1/3, 2/3, 1] with the values [0, $n_{1}$, $n_{1} + n_{2},$1] times the maximal radius $r_{max}$, which is defined by *a*. The trajectory equation for $k_{traj} = k_{x} + 1ik_{y}$ with $1i$ the imaginary number is stated below:

$$k_{traj}=r_{max}\cdot r(t, n_{1},n_{2})\cdot exp(-2\pi i{(v_{1}t)}^{\frac{1}{v_{2}}})$$

with $t\in\{0, \frac{1}{s-1}, \frac{2}{s-1},\ldots, 1\}$

**Supporting Information Eq. S 2**


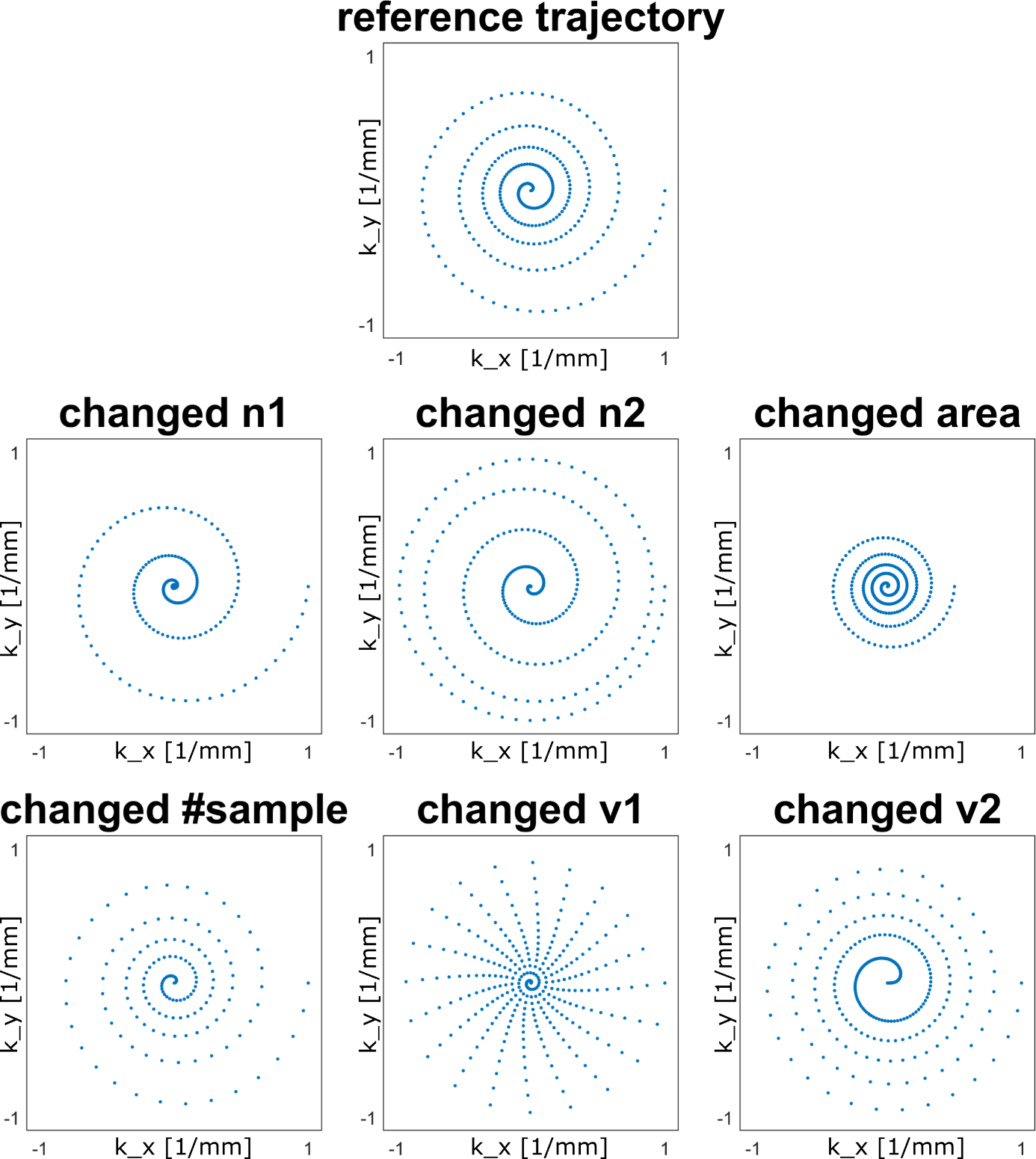


**Supporting Information Fig. S 1** Illustration of the influence of the individual parameters on a reference trajectory. The nodes $n_{1}$ and $n_{2}$ determine how fast the spiral converges towards the center. The coverage of k-space is influenced by parameter *a* (here: reduction by a factor 2). General density can be changed by the number of samples (here: reduction by a factor of 2). A kind of sampling rate is implemented with factor $v_{1}$. The change of this sampling rate can be controlled with $v_{2}$. For all mappings, the parameters except the one mentioned in the subtitles are kept as for the reference trajectory

The optimization procedure involves the generation of the trajectory based on ten initial randomly selected parameter sets (each consisting of the six parameters introduced in Supporting Information Eq. S 2) as start values and a subsequent RF pulse calculation as described in the main manuscript’s methods for all six volunteer data sets. The suitability of the found COV is expressed by a cost function (Supporting Information Eq. S 3), which consists of the deviation between the target FA map ${FA}_{tar}$ (i.e. 110°/0° for fat/water) and a Bloch simulated FA map of the SPSP pulse calculation ${FA}_{act}$ for all volunteer data sets $N_{V}$ and a penalty, when slew-rate constraints are violated. The iterative optimization is performed according to the methods of the patternsearch algorithm provided by MATLAB as part of the Global Optimization Toolbox in R2019b (The MathWorks, Natick, MA). The workflow is illustrated in Supporting Information Fig. S 2.

$$cost :=\sum_{N_{V}=1}^{6} \left( 1+P_{SR, N_{V}} \right)\cdot exp({NRMSE}_{\alpha,N_{V}})$$

**Supporting Information Eq. S 3**

where $P_{SR,N_{V}}\in\{0,1\}$ is an additional penalty per volunteer, ensuring that the slew-rate boundaries set by the system are met. ${NRMSE}_{\alpha,N_{V}}=\sqrt{\frac{1}{N\cdot F}\sum_{f=1}^{F} \sum_{n=1}^{N_{voxel}} {({FA}_{act}^{n,f}-{FA}_{tar}^{n,f})}^{2}}$ represents the normalized root mean square error, i.e. the deviation between the actual simulated and target FA maps (for all frequencies $F$ and voxels $N_{voxel}$) at 0.0 ppm and ‑3.4 ppm of one volunteer. Since the optimization problem is non-convex, several runs with different initial points are applied.


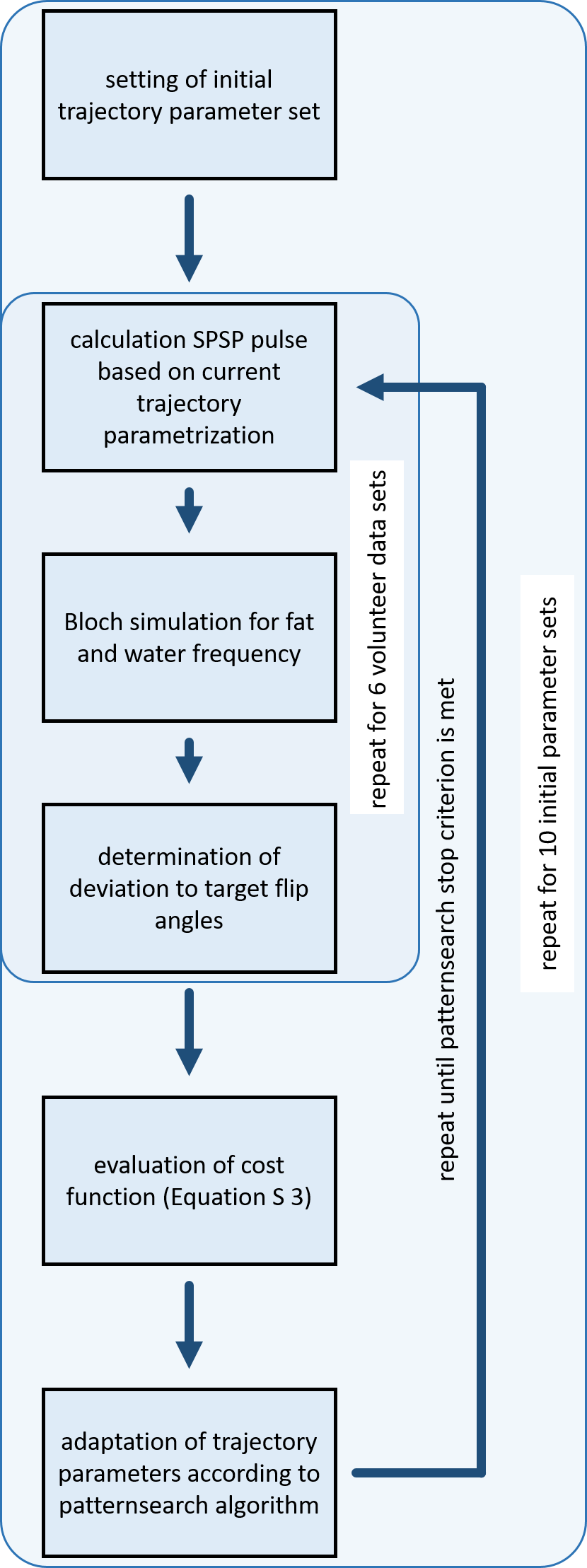


**Supporting Information Fig. S 2** Offline trajectory optimization workflow specified for six volunteer data sets and ten initial points. The stop criterion of the patternsearch algorithm is related to the stepsize of the adaptation of the trajectory parameters

MATLAB’s parallel computing toolbox enables simultaneous optimization within one iteration on multiple cores on a desktop computer (Intel Core i5-8400; CPU 2.80 GHz; 6 Kernels; RAM 16 GB).

The lower and upper boundaries for the optimization procedure are given in Tab. S 1. The whole optimization process takes ten days. Afterwards the COV with the lowest final cost function is selected as final COV set (Tab. S 1, last column). The absolute maximum spatial frequency (maximum radius) in k-space is 0.32 cm^-1^ (see Fig. 1, main manuscript). Therefore, the minimum pixel width is $\text{∆w}\text{ }\text{=}\text{ }\frac{\text{1}}{\text{0.32}}\text{ cm}\text{ }\text{=}\text{ }\text{3.13}\text{ }\text{cm}$. The trajectory can be divided into an outer spiral (between 0.26 cm^-1^ and 0.32 cm^-1^), a transition part and an inner spiral (between k-space center and 0.06 cm^-1^). The outer spiral is sampled for 3.2 ms and covers relatively high spatial resolutions of 3.13-3.85 cm. Since B0 inhomogeneities are usually spatially smooth with low spatial frequencies, the inner spiral (4.2 ms) covers low spatial resolutions of 16.0 cm or more. Fig. 1 b of the main manuscript shows that the mayor part of the RF power is applied here. The overall simulated FAs for all six volunteer data sets used in the optimization are 108.6° ± 2.5° for fat and 4.5° ± 1.9° for water. Comparable results are obtained for the six data sets only used for evaluation with mean FAs of 108.3° ± 3.3° for fat and 4.8° ± 1.8° for water.

**Tab. S 1** Lower and upper boundaries for the COV of the defined excitation k-space trajectory with temporally alternating radius and velocity in k-space. Last column represents the final COV that is used for all further experiments.$:=[n_{1}, n_{2}, a, s, v_{1}, v_{2}]$

|  | **lower boundary** | **upper boundary** | **final COV** |
| --- | --- | --- | --- |
| node 1 ($n_{1}$) | 0 | 1 | 0.01 |
| node 2 ($n_{2}$) | 0 | 1 | 0.99 |
| k-space area ($a$) [%] | 0.05 | 0.95 | 0.36 |
| # samples ($s$) | 500 | 1050 | 990 |
| k-space velocity factor 1 ($v_{1}$) | 0 | 5000 | 500 |
| k-space velocity factor 2 ($v_{2}$) | 1 | 5 | 1.85 |

## Comparison to standard 2D spiral and 2D spline-interpolated trajectory

The proposed trajectory design is a suitable choice for the targeted fat saturation pulse calculation. However, since the general optimization problem is assumed to have a high number of local minima, it is likely that the found parameters and also the trajectory design itself do not represent the best solution, i.e. the global minimum. To show that the final 2D spiral trajectory and the previously discussed optimization procedure improve the performance of the proposed pulse design, we compare the tailored trajectory with a standard 2D spiral trajectory and a 2D spline-interpolated trajectory (see Supporting Information Fig. S 3 a). The k-space coverage is chosen similarly to the tailored trajectory. In general, this parameter would have to be determined separately, e.g. by experience or another optimization method. For the 2D spiral trajectory, the number of equidistant revolutions is chosen randomly between 5 and 30 (result: 13). The 2D spline-interpolated trajectory is created by selecting 21 ordered (from outer excitation k-space to the center) and random points in k-space, which are interpolated using MATLAB’s 1-D data interpolation with the "spline" option.

The comparison is conducted with the help of individual pulse calculations based on the 12 volunteer data sets used for optimization and evaluation of the proposed trajectory. The mean flip angle errors to the targeted FA (i.e. 110° for fat, 0° for water) are shown in Tab. S 2 and the corresponding boxplots in the Supporting Information Fig. S 3 b.

**Tab. S 2** FA errors at fat and water frequencies for individual pulses from 12 volunteer data sets based on the proposed spiral, a standard spiral and a spline-interpolated trajectory. The parameters for the latter two are chosen randomly, while the proposed spiral trajectory is optimized as described above

| FA error [°] | **proposed spiral** | **standard spiral** | **spline-interpolated** |
| --- | --- | --- | --- |
| fat | 1.6 ± 2.0 | 10.6 ± 3.7 | 36.8 ± 6.4 |
| water | 4.6 ± 1.3 | 11.6 ± 2.5 | 34.5 ± 5.9 |


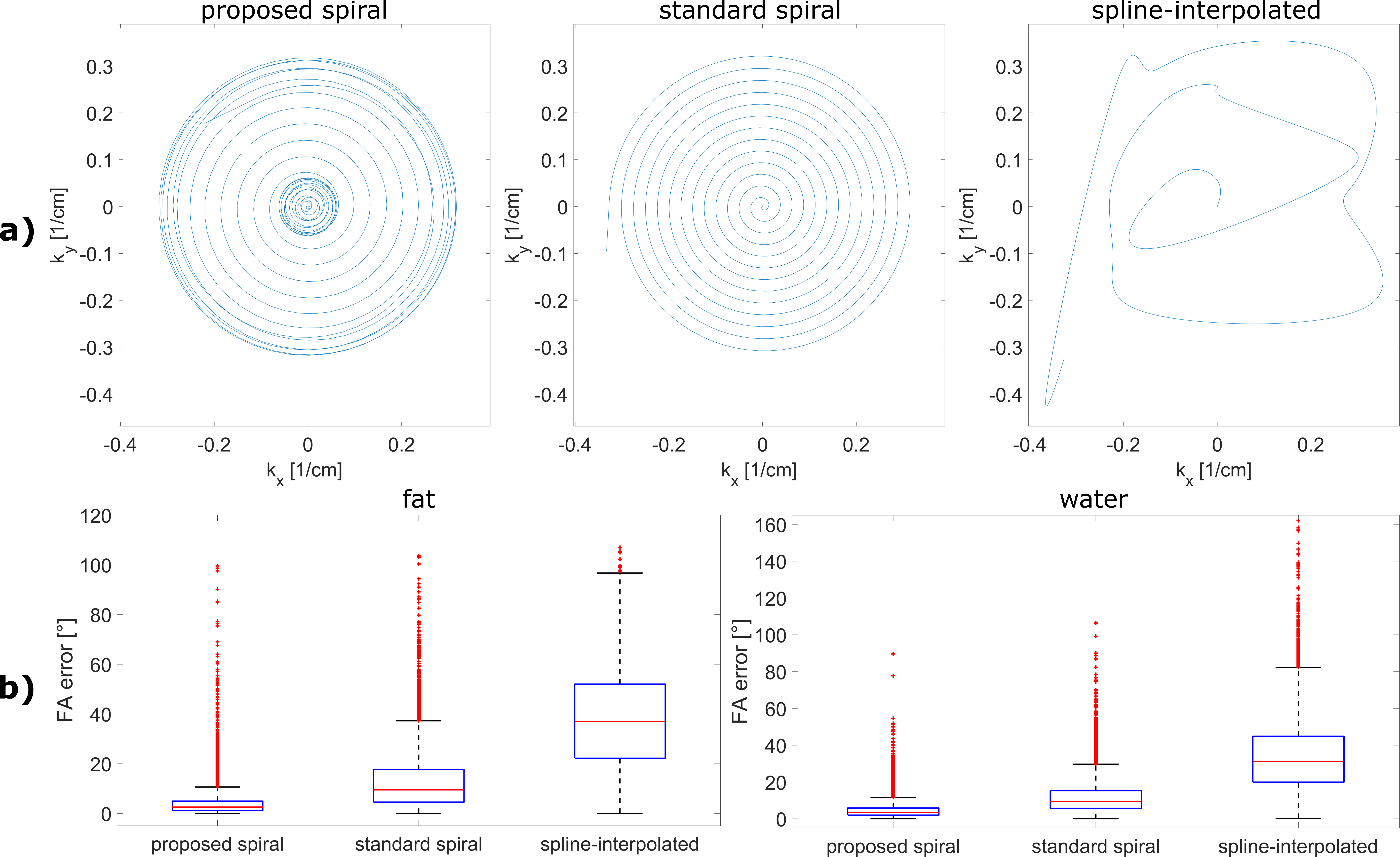


**Supporting Information Fig. S 3** Comparison of the proposed spiral trajectory with a standard spiral trajectory and a spline-interpolated trajectory with similar excitation k-space coverage (trajectories shown in a). Quantitative analysis of the resulting FA errors at fat and water frequency for 12 volunteer data sets shows that the tailored trajectory outperforms the other approaches with non-optimized parameters. The simulated flip angle errors to the target flip angles (i.e. 110° for fat, 0° for water) of all voxels and volunteers are presented in b)

# Further investigations of the proposed method

## All qualitative volunteer measurements: sagittal foot/ankle

In this section, a more detailed presentation of sagittal foot and ankle measurements is given to demonstrate a possible extension of the proposed method beyond sagittal cervical spine imaging. Fig. 2 c) of the main manuscript shows an exemplary foot/ankle measurement of one volunteer. The qualitative results of all five volunteer measurements are shown in Supporting Information Fig. S 4. For better illustration, only the "SPSP standard" configuration is included. The effects of the accelerated configurations are presented exemplarily in Fig. 2 c) of the main manuscript. All five volunteers show similar results for these configurations.


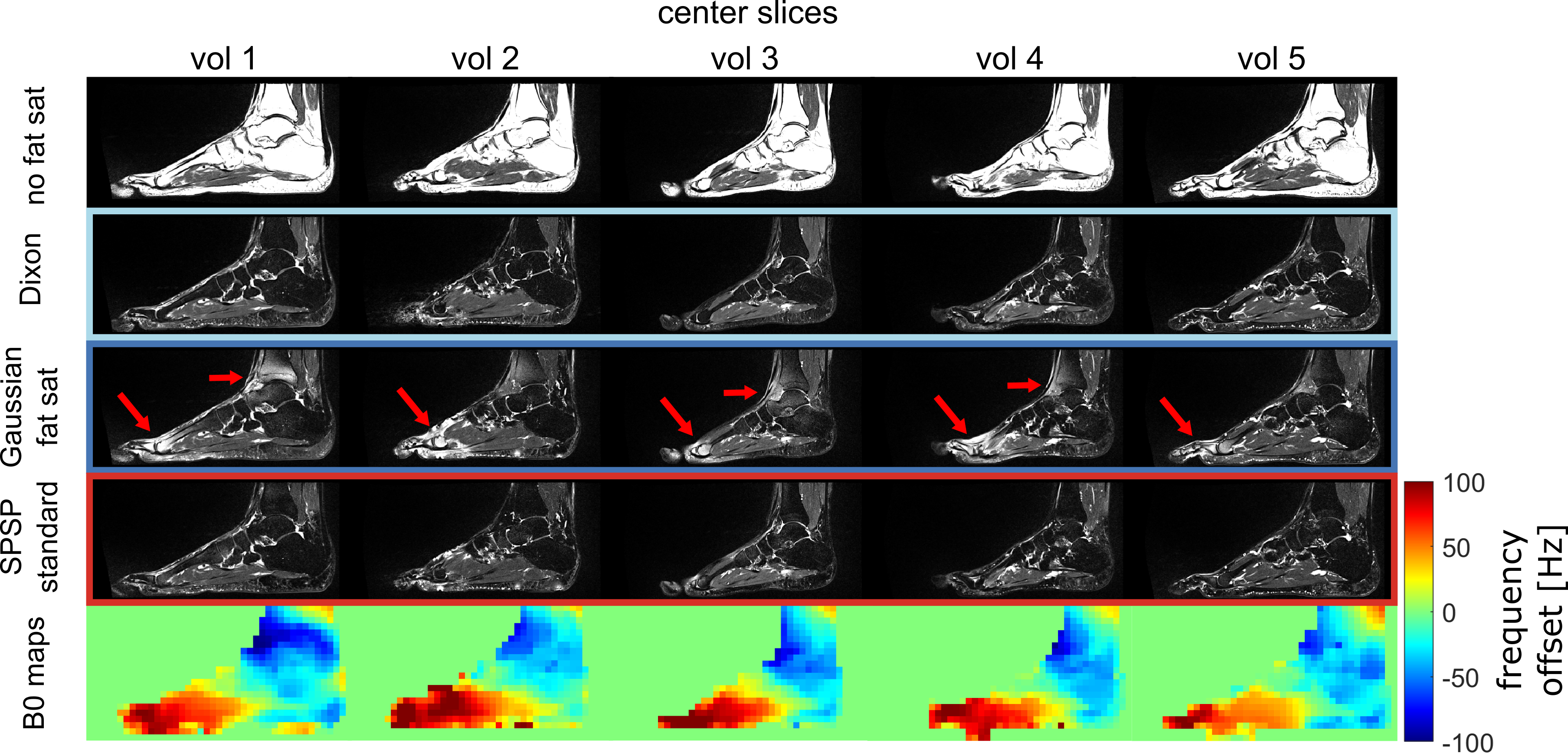


**Supporting Information Fig. S 4** Extension of Fig. 2 c) of the main manuscript, where only the results of one volunteer (here: vol 5) are shown. All five volunteer data sets demonstrate similar behavior, with insufficient fat saturation towards the toes and ankle (red arrows) for the Gaussian fat saturation pulse. The proposed SPSP method improves fat saturation by including B0 information in the pulse calculation

## Outlook on other image orientations

The focus of this work is to demonstrate the technical feasibility of the proposed method. A suitable body region and orientation for this is the sagittal cervical spine, as B0 inhomogeneities are in the range of the fat-water frequency difference. Therefore, a standard spectral fat saturation approach is likely to fail in this region and orientation. Furthermore, we demonstrate the transferability of the method to sagittal foot/ankle imaging by measurements and to other body regions and orientations by simulations (see main script).

Supporting Information Fig. S 5 shows measurements of the proposed method (configuration SPSP bfgs500 sc2) for coronal foot/ankle (a, male, 45 years) and transversal cervical spine (b, male, 54 years) imaging. The protocol parameters are TR = 2450 ms, TE = 38 ms, TA = 2:44 min, FOV = (140.0x114.2x73.7) mm^3^, resolution = (0.5x0.5x2.1) mm^3^, slices = 32 and TR = 3850 ms, TE = 84 ms, TA = 3:14 min, FOV = (250.0x250.0x43.2) mm^3^, resolution = (0.4x0.4x3.0) mm^3^, slices = 11, Deep Resolve Gain and Sharp for foot/ankle and cervical spine measurements, respectively. For the calculation of the SPSP pulses, the same settings and workflow (including the same tailored trajectory) are used as for all other experiments.

The coronal foot/ankle images appear similar, although the fat signal is slightly less suppressed for the proposed method (highlighted by red arrows) than for the Gaussian fat saturation method. A possible explanation for this is the low resolution of the B0 map, which is downscaled by a factor of four. This setting is chosen to reduce the optimization problem and thus also the calculation time. However, for small FOVs and more detailed anatomical structures, such as in the coronal foot/ankle region, downscaled field maps might not be accurate enough.

SPSP pulses lead to greatly improved fat saturation in the transversal cervical spine images. The Gaussian fat saturation pulse excites water in areas where the B0 offset shifts the water frequency to the nominal fat frequency. These areas (marked with red arrows) represent fat-only contrast instead of being fat-suppressed. The proposed method accounts for these offsets and is able to maintain the desired contrast.

Since these measurements are performed with only one volunteer each, this is not a general proof of the applicability of the proposed method (e.g. the tailored trajectory) to the whole body and all orientations. Some measurements may require a change in the configuration of the pulse calculation and/or an adaptation of the trajectory.


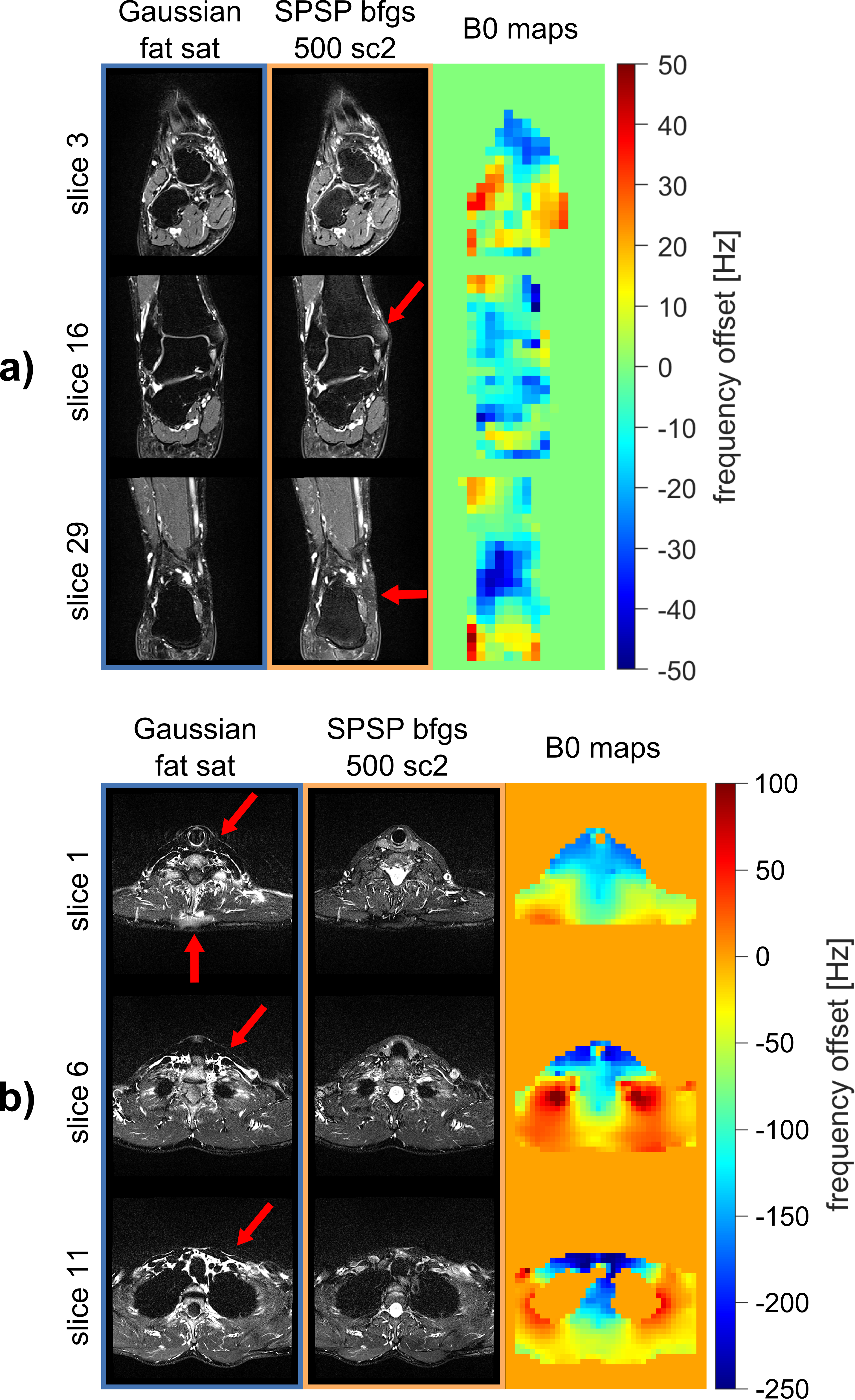


**Supporting Information Fig. S 5** Measurements of a) coronal foot/ankle and b) transversal cervical spine fat saturated images with Gaussian and SPSP pulses. Coronal foot/ankle images for both methods appear similar, with slightly more fat remaining with the SPSP approach (marked with red arrows). Transversal cervical spine images with Gaussian fat saturation show strong water excitation in some areas (marked with red arrows) instead of fat saturation due to strong B0 offsets. SPSP pulses are able to compensate for these inhomogeneities, resulting in more homogeneous fat saturation

## Universal SPSP pulse design

This work focuses on individually designed SPSP pulses. In addition, a universal pulse is determined based on the average of the individual pulses of the six data sets used for trajectory optimization. This pulse is applied to the data of the five in vivo measurements in simulations. The performance of an exemplary volunteer at fat and water frequency compared to the individual pulse is shown in Supporting Information Fig. S 6 together with a quantitative summary of all five data sets. The individual SPSP pulses outperform the universal pulse approach at both target frequencies. Simulations show that the universal pulse leads to mean deviations from the target flip angles of fat and water of (33.8 ± 17.9)° / (19.4 ± 11.4)°, respectively. The mean deviations across all slices and volunteers for the individual pulses are (5.9 ± 8.0)° / (6.1 ± 7.5)°. However, there are more sophisticated approaches to design a universal pulse, e.g. concatenating all data sets for pulse optimization instead of averaging the results of the individual optimizations [1,2], which could improve the expected fat saturation quality.

**Supporting Information Fig. S 6** Comparison of individual and universal pulse design, where the universal pulse is calculated as average of the individual pulses of the trajectory optimization data sets. An exemplary data set shows the differences at a) fat and b) water frequency in three slices. The corresponding B0 maps are presented in c). Quantitative analysis of simulations d) of the five in vivo data sets from section 3.2 of the main manuscript illustrates the expected performance of both approaches


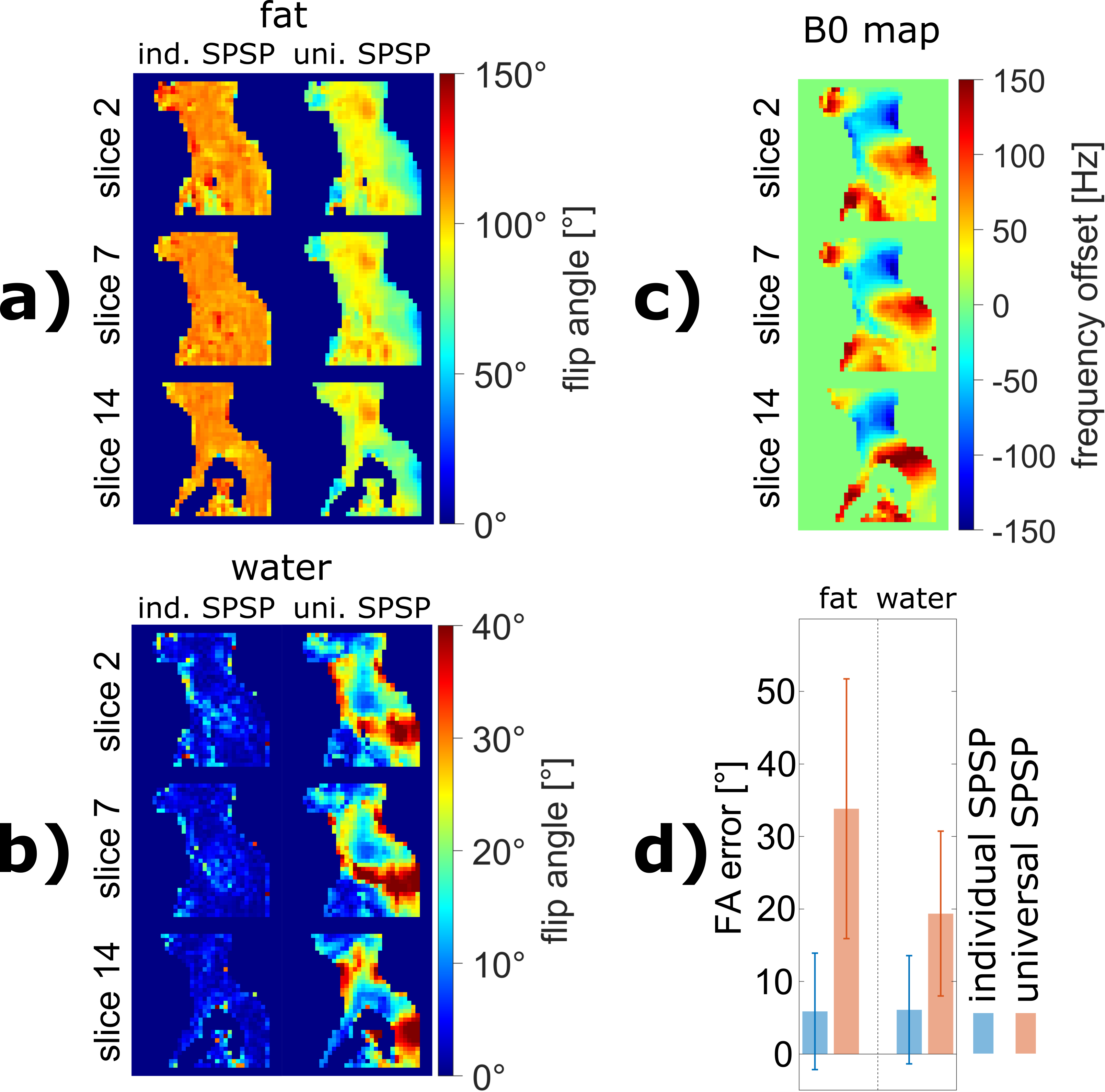


## Impact of the slice block size in multi-slice application

Since the selected trajectory includes only in-plane gradients, the pulse design described in section 2.2 of the main manuscript focuses on the optimization of a single slice. For multi-slice acquisitions, one SPSP pulse per slice must be determined independently. This results in a strongly increased preparation time (by a factor of the number of slices), which is why we seek to apply one common pulse to multiple slices that have a similar B0 distribution. This similarity is defined by the requirements stated in Eq. 2 of the main manuscript. The common pulse is determined based on the center slice of the block of slices that are similar. This reduces the preparation time as fewer pulses need to be optimized.

It is expected that the performance of the common pulse on slices more distant from the center slice decreases depending on the size of the slice block. This effect is investigated in the five in vivo sagittal cervical spine data sets of section 3.2 for three different slice block sizes (1 pulse per 1/5/15 slices) in simulations. The pulses for the slice blocks are calculated based on each slice (15 pulses), slices 3, 8 and 13 (3 pulses) and slice 8 (1 pulse). The qualitative results for every second slice of an exemplary data set are shown in Supporting Information Fig. S 7. In addition, the figure shows the quantitative analysis for all five data sets. The slices on which the corresponding SPSP pulse calculation is performed are marked with an asterisk.

The qualitative and quantitative results illustrate the lower performance for slices that are not used for optimization. In general, the greater the distance to the center slice of a slice block, the greater the differences in the B0 maps. Therefore, the solution based on the center slice fits the given offsets less well and the deviations from the target flip angles increase. This effect is particularly visible in the case where only one pulse is determined for all 15 slices. For slice 8, the performance is the same as for the other settings ((1.0 ± 3.6)° / (3.2 ± 2.6)° for fat and water, respectively), as all pulses are determined on the basis of this slice. However, the performance for the most distant slices drops to (4.4 ± 11.5)° / (7.7 ± 8.1)° (slice 1) and (4.0 ± 14.6)° / (9.2 ± 10.1)° (slice 15). The potential reduced quality of the single slices depends on the differences of the B0 distributions and the threshold values that determine the slice blocks.


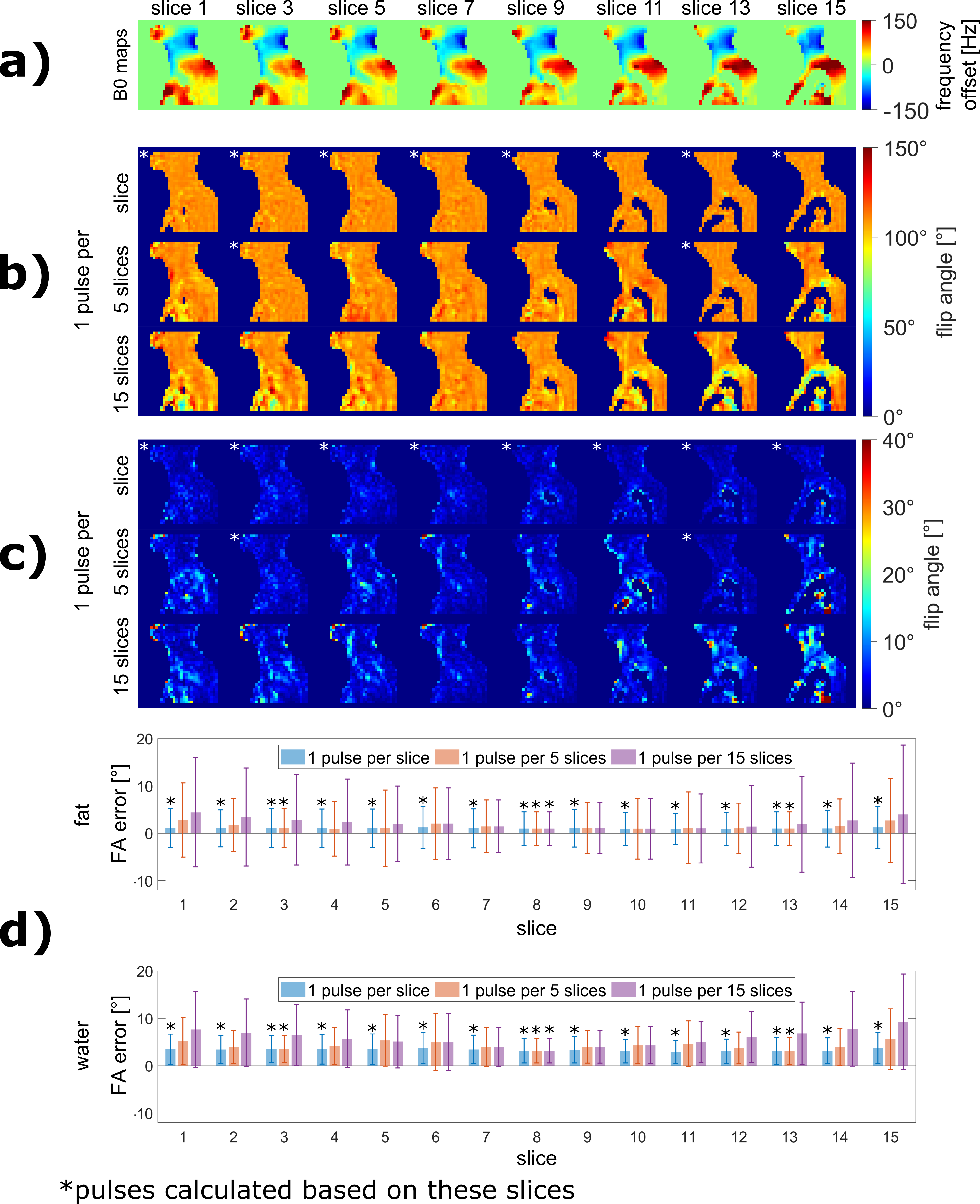


**Supporting Information Fig. S 7** Simulations on the impact of the size of slice blocks that share a common pulse. For the proposed SPSP fat pre-saturation method, the slice blocks are automatically determined based on Eq. 2 of the main manuscript. For this analysis, the slice blocks are set to 1 pulse per 1/5/15 slices. a) B0 maps, results at b) fat and c) water frequencies for an exemplary data set are presented. The fewer pulses are determined, the more the performance in the individual slices decreases. This can also be observed in d) the quantitative results of all five in vivo data sets, where the FA error to the target state is shown as function of the slices

# References

1. Herrler J, Liebig P, Gumbrecht R, Ritter D, Schmitter S, Maier A, Schmidt M, Uder M, Doerfler A, Nagel AM (2021) Fast online-customized (FOCUS) parallel transmission pulses: A combination of universal pulses and individual optimization. Magn Reson Med 85 (6):3140-3153.

2. Gras V, Vignaud A, Amadon A, Le Bihan D, Boulant N (2017) Universal pulses: A new concept for calibration-free parallel transmission. Magn Reson Med 77 (2):635-643.
